# Physician and Hospital Performance in Medicare’s Updated Bundled-Payment Model for Joint Replacement

**DOI:** 10.1001/jamahealthforum.2025.1930

**Published:** 2025-07-25

**Authors:** Aidan P. Crowley, Austin S. Kilaru, Qian (Erin) Huang, Erkuan Wang, Jingsan Zhu, Ayush Arora, Torrey Shirk, Deborah S. Cousins, Kristin A. Linn, Said A. Ibrahim, Joshua M. Liao, Amol S. Navathe

**Affiliations:** 1 The Parity Center, Department of Medical Ethics and Health Policy, Perelman School of Medicine, University of Pennsylvania, Philadelphia; 2 Leonard Davis Institute of Health Economics, University of Pennsylvania, Philadelphia; 3 Department of Health Care Management and Economics, The Wharton School, University of Pennsylvania, Philadelphia; 4 Department of Emergency Medicine, Perelman School of Medicine, University of Pennsylvania, Philadelphia; 5 Editorial Fellow, *JAMA Health Forum*; 6 Sidney Kimmel Medical College, Thomas Jefferson University, Philadelphia; 7 Department of Biostatistics, Epidemiology, and Informatics, Perelman School of Medicine, University of Pennsylvania, Philadelphia; 8 Associate Editor, *JAMA Health Forum*; 9 Department of Internal Medicine, University of Texas Southwestern Medical Center, Dallas

## Abstract

**Importance:**

Independent evaluations of Bundled Payments for Care Improvement Advanced (BPCI-A) have focused on hospitals and have not assessed the performance of physicians in participating physician group practices (PGPs). However, PGPs are accountable for a larger proportion of surgical procedures, including for lower-extremity joint replacement, in the BPCI-A model than are hospitals.

**Objective:**

To evaluate the association of treatment by BPCI-A−participating physicians and hospitals with health care spending, quality, and utilization for joint replacement procedures compared to nonparticipants.

**Design, Setting, and Participants:**

This cohort study used Medicare claims of beneficiaries receiving lower-extremity joint replacement between April 2016 and September 2019 and data on BPCI-A−participating PGPs and hospitals to assess spending, quality, and utilization. Differences-in-differences methods adjusting for patient and market characteristics (aDID) were used with matched comparison groups of nonparticipating physicians and hospitals. Data analysis was performed from January 2023 to January 2025.

**Exposures:**

Lower-extremity joint replacement by a physician in a PGP or hospital that began BPCI-A participation in October 2018.

**Main Outcomes and Measures:**

Ninety-day total episode spending for joint replacement. Secondary outcomes were postacute care utilization, mortality, hospital readmissions, and joint replacement complications.

**Results:**

The matched cohort included 846 529 Medicare beneficiaries (mean [SD] age, 73.7 [8.3] years; 63.8% female) who obtained a joint replacement in April 2016 to September 2019, of whom 281 189 were treated by 2820 physicians in BPCI-A−participating PGPs, and 69 107 by 174 BPCI-A−participating hospitals. An additional 28 309 beneficiaries were treated by physicians and hospitals both participating in BPCI-A. The remaining 467 924 were treated by 4671 nonparticipating physicians and 432 nonparticipating hospitals. Before BPCI-A participation, total unadjusted baseline episode spending was $26 483 for participating physicians and $29 854 for participating hospitals. Treatments by BPCI-A participating physicians and hospitals were each associated with differentially lower total spending (physician aDID, −$855; 95% CI, −$1074 to −$636; hospital aDID, −$613; 95% CI, −$1039 to −$187). Treatment by a BPCI-A−participating physician or hospital was associated with differentially lower institutional postacute care utilization. Physician participation was associated with a differential increase in outpatient visits 7 days postdischarge (aDID, 2.9 percentage points; 95% CI, 2.0 to 3.8), while hospital participation was not associated with a change in outpatient visits. Differential changes in mortality, readmissions, and complications were not observed for either participant type.

**Conclusions and Relevance:**

This cohort study found that participation in BPCI-A for joint replacement was associated with differentially lower total spending for both physicians and hospitals. Given that physicians in PGPs accounted for 73% of all the joint replacement episodes, these findings highlight the importance of facilitating alignment between hospitals and physicians in future bundled-payment models, including those that allow only hospitals.

## Key Points

**Question:**

Are physician and hospital participation in the Medicare Bundled Payments for Care Improvement Advanced (BPCI-A) associated with changes in health care spending, quality, and utilization for lower-extremity joint replacement?

**Findings:**

This cohort study including 846 529 Medicare beneficiaries found that treatment by physicians in BPCI-A−participating group practices was associated with an $855 differential reduction in spending at 90 days compared to nonparticipating practices. Likewise, treatment at BPCI-A−participating hospitals was associated with a $613 differential reduction.

**Meaning:**

These findings suggest that physicians and hospitals may be effective at reducing spending in bundled payments, and that future models should consider strategies to align physicians when participation is restricted to hospitals.

## Teaser

This cohort study evaluates treatment by physicians and hospitals participating in Medicare’s Bundled Payments for Care Improvement Advanced and changes in spending, quality, and utilization for joint replacement procedures.

## Introduction

Episode-based payment models are a key strategy to improve quality and cost-efficiency for patients with surgical conditions or acute medical illness.^1,2^ The US Centers for Medicare & Medicaid Services (CMS) have tested several episode-based models, commonly known as bundled payments.^3^ Bundled payments have consistently demonstrated reduced spending for surgical episodes, particularly orthopedic procedures, with mixed results for medical episodes.^2,4,5,6,7,8,9,10,11,12,13,14,15^ CMS has extended its current voluntary bundled-payment model, Bundled Payments for Care Improvement Advanced (BPCI-A), through the end of 2025.^16^

In model years 1 and 2 (October 2018-December 2019), hospitals or physician group practices (PGPs) elected to participate in BPCI-A for any of 32 clinical conditions. Participants were accountable for spending within 90 days of an index admission and retained any savings or sustained any losses relative to a target price. PGPs have been, by far, the most common participant in episodes for lower-extremity joint replacement surgery (hereafter, joint replacement) compared to hospital participants.^17^ However, independent evaluations of the model have focused on hospitals.^4,6,7,15,18,19^ Evidence from prior models suggests that PGPs use different strategies than hospitals to reduce spending, including the shifting of postacute care toward physician visits rather than home health, producing differences in model performance.^20,21^

CMS has announced a new mandatory bundled-payment model, Transforming Episode Accountability Model (TEAM), which will begin in 2026.^22^ TEAM will focus on 5 surgical conditions, including joint replacement. Only hospitals, and not PGPs, will be eligible to participate in the model. Given that TEAM will be mandatory for more than 700 hospitals located in 25% of US markets, evaluating performance differences by participant type in prior models, including BPCI-A, may provide insight into the impact of restricting participation to hospitals despite program differences. Furthermore, TEAM may permit gainsharing, in which a participating hospital can share a portion of incentive payments with the physician who performs the procedure. If physicians have demonstrated strong performance under prior bundled-payment models, albeit voluntary, this may provide empirical support for TEAM to more proactively align incentives between hospitals and physicians through gainsharing.^23^

The objective of this study was to evaluate the association of physician and hospital participation in BPCI-A with health care spending, quality, and utilization in joint replacement episodes compared to nonparticipating physicians and hospitals.

## Methods

The University of Pennsylvania Institutional Review Board approved this study. Informed consent was waived because the research involved no more than minimal risk; patients for whom records were reviewed were not readily available to sign a consent; a possibility existed of creating additional risks to privacy by linking otherwise deidentified data with nominal identifiers to contact individuals to seek consent; and the waiver did not adversely affect the rights and welfare of the participants. This study followed the Strengthening the Reporting of Observational Studies in Epidemiology (STROBE) reporting guideline.

### Data Source and Study Population

We conducted a retrospective cohort study to determine the impact of participation in BPCI-A on Medicare beneficiaries who received joint replacement. We used a 100% file of Medicare claims data to identify all fee-for-service beneficiaries with an inpatient admission for joint replacement (Diagnosis Related Groups [DRGs] 469 and 470) from April 2016 to September 2019. We identified patient characteristics using the Master Beneficiary Summary File. We excluded episodes for beneficiaries who were not continuously enrolled in Medicare, were enrolled in Medicare Advantage (MA), or had a primary payer other than Medicare (eMethods 1 in Supplement 1). Per program rules, we excluded beneficiaries with end-stage kidney disease or who died during the index admission.

We identified physician characteristics from the Medicare Data on Provider Practice and Specialty, National Plan and Provider Enumeration System, and Physician Compare files. We identified hospital characteristics from American Hospital Association data and hospital spending from the Medicare Provider Analysis and Review file. We identified market-level characteristics from the American Community Survey and the Provider of Services file.

We included hospitals and PGPs with at least 1 joint replacement episode in both the pre- and postperiod. Following prior work, we excluded hospitals that took the one-time opportunity to withdraw from the program in March 2019 from both intervention and comparison groups.^19^ Inclusion and exclusion criteria are further detailed in the eMethods 1 in Supplement 1.

### Study Groups

We used BPCI-A participation and reconciliation files to identify hospitals and physicians that participated in the model from October 2018 to September 2019 (model years 1-2).^24,25^ We used this file to identify hospitals participating in BPCI-A for joint replacement and linked these to hospital CMS Certification Numbers (CCNs). To identify participating physicians, we linked National Provider Identifiers in the participation files to claims in which either the attending or operating physician was listed for the index admission (eMethods 2 in Supplement 1).^21^ We defined nonparticipants as hospitals or physicians that did not participate in BPCI-A for any condition in the orthopedic clinical episode service-line group (eMethods 3 in Supplement 1).

### Episode Construction

To construct bundled-payment episodes, we identified joint replacement claims eligible for BPCI-A using DRGs 469 and 470, combining all claims associated with the index admission. We applied a 90-day nonoverlapping rule by assigning overlapping episodes (for joint replacement or any other BPCI-A−eligible condition) to the earlier hospitalization.^21,26^ Then, we added all claims within 90 days of discharge from the index admission and excluded certain claims based on program rules (eMethods 1 in Supplement 1). We included 1 random episode per beneficiary during the study period.

### Outcomes

The primary outcome was 90-day total joint replacement episode spending, calculated as the sum of hospital spending for the index stay and all spending through 90 days postdischarge. Secondary outcomes were 30-day total episode spending and spending on episode components, including the index admission, readmissions, postacute care, emergency department (ED) visits, and professional services. Following prior methods, we standardized spending to allow for comparison across physicians and hospitals and geographic areas (eMethods 4 in Supplement 1).^27^ Secondary outcomes also included quality and utilization at 30 and 90 days. Quality measures included mortality, all-cause readmissions, ED visits, and joint replacement complications.^28^ Utilization measures included postacute care as well as outpatient physician visits within 7 days of discharge from the index hospitalization.

### Variables

The primary exposure variable was joint replacement by a physician and/or a hospital that began participating in BPCI-A in October 2018 (eMethods 5 in Supplement 1). We adjusted for the following patient characteristics: sex, race and ethnicity, age, disability status, Medicare-Medicaid dual eligibility, Elixhauser Comorbidity Index, prior hospitalizations, prior use of postacute care, and an indicator for hip surgery vs knee surgery. Race and ethnicity were assessed according to the CMS beneficiary race and ethnicity code and categorized as Black, White, or other (including Asian, Hispanic, North American Native, other, and unknown). At the BPCI-A participant level (ie, physician or hospital), we included indicators for participation in prior bundled-payment programs for joint replacement (BPCI or Comprehensive Care for Joint Replacement). We adjusted for market-level characteristics, including number of Medicare beneficiaries, volume of joint replacement episodes, Accountable Care Organization penetration, MA penetration, skilled nursing facility (SNF) concentration (Herfindahl-Hirschman Index), SNF beds per 10 000 patients, and hospital beds per 10 000 patients. For Accountable Care Organization penetration, MA penetration, hospital concentration, and Elixhauser Comorbidity Index, we included interaction terms with time (quarter-years) to account for time-varying effects on outcomes, following prior literature.^21^

### Statistical Analysis

We used difference-in-differences methods to compare changes in outcomes for Medicare beneficiaries receiving care before compared with after initiation of BPCI-A from participants vs from propensity score−matched nonparticipants. Following prior methods and because physicians and hospitals chose BPCI-A participation separately (ie, a patient can receive surgery from a physician in a participating PGP at a nonparticipating hospital or vice versa, or any combination of the 2), we used a quasi-experimental design that emulated a 2 × 2 factorial randomized trial in which patients received care from 1 of 4 mutually exclusive groups: physicians in BPCI-A−participating PGPs, BPCI-A−participating hospitals, both, or neither.^21^

We used greedy 3:1 propensity score matching with replacement to identify comparison groups of nonparticipating hospitals and physicians. We matched physicians at the National Provider Identifier level using physician, practice, and market-level characteristics with an exact match on core-based statistical area type (metropolitan, micropolitan, or rural). We matched hospitals according to hospital characteristics, joint replacement volume, and market-level characteristics with an exact match on teaching status (eMethods 6 in Supplement 1). We assessed the results of the matching procedure through standardized mean differences (eTables 1 and 2 in Supplement 1).

We modeled primary and secondary outcomes using a generalized linear model with time, hospital, and DRG fixed effects (eMethods 7 in Supplement 1). We used cluster-robust standard errors, clustered at the hospital level. Holm-Bonferroni adjustment was applied to *P* values for the primary outcome of 90-day spending to correct for multiple testing. Examination of preintervention parallel trends did not suggest divergence (eFigure in Supplement 1).

### Sensitivity and Exploratory Analyses

We conducted 6 sensitivity analyses. First, we repeated the main analysis using a 1:1 matching approach. Second, we repeated the analysis treating the *both* group as the physician group to align with program rules that assign precedence to PGP over hospital participants. Third, we aggregated all 3 BPCI-A groups (physician only, hospital only, and both) to report an overall program effect. Fourth, we excluded quarters 2 and 3 (April to September) of 2018 as a washout period to avoid anticipation effects. Fifth, we assessed the accuracy of the factorial design by analyzing hospital and physician participation effects separately and excluding episodes under the opposite group. Sixth, we restricted the control group to hospitals that did not participate in BPCI-A at all, regardless of service-line group.

We also conducted exploratory analyses to identify potential evidence of selective participation in BPCI-A. We modeled each of the following: mean Elixhauser Comorbidity Index, share of dual-eligible beneficiaries, share of disabled beneficiaries, share of beneficiaries with prior SNF use, and share of beneficiaries with prior hospitalizations over time to explore whether these changed differentially between participants and nonparticipants. An additional exploratory analysis assessed unadjusted outcomes in the 31- to 90-day period to describe outcomes for the final 60 days of episodes. Statistical tests were 2-tailed and *P* < .05 was considered statistically significant. Data analyses were performed from January 2023 to January 2025 using SAS, version 9.4 (SAS Institute).

## Results

**Figure 1.**
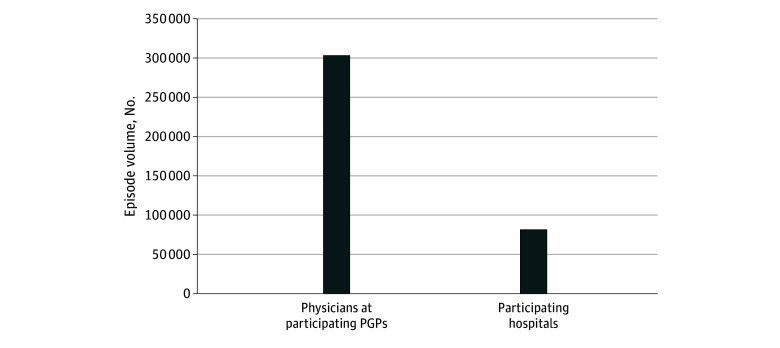
Total Volume of Joint Replacement Episodes Performed by Physician Group Practices (PGPs) and Hospitals Participating in Medicare Bundled Payments for Care Improvement Advanced

The matched cohort included 846 529 Medicare beneficiaries (mean [SD] age, 73.7 [8.3] years; 63.8% female and 36.2% male) who obtained a joint replacement from April 2016 to September 2019 (Table 1). Of these, 281 189 beneficiaries received joint replacement from 2820 physicians in BPCI-A−participating PGPs, and 69 107 from 174 BPCI-A−participating hospitals. An additional 28 309 beneficiaries received the procedure from BPCI-A−participating physicians and hospitals, ie, both participating (Figure 1). The remaining 467 924 beneficiaries received joint replacement from 4671 nonparticipating physicians and 432 nonparticipating hospitals (Table 1).

**Table 1.** Characteristics of Medicare Beneficiaries, by Bundled Payments for Care Improvement Advanced (BPCI-A) Participants vs Nonparticipants (NP), Before and After October 2018

Characteristic	Beneficiaries, No. (%)							
	BPCI-A physician only		BPCI-A hospital only		BPCI-A both		NP	
	Before	After	Before	After	Before	After	Before	After
Total beneficiaries, No.	201 906	79 283	51 302	17 805	20 678	7631	344 493	123 431
Age, mean (SD), y	73.7 (8.1)	73.9 (8.0)	73.5 (8.7)	73.9 (8.7)	73.7 (8.3)	74.1 (8.0)	73.7 (8.4)	73.9 (8.3)
Sex								
Female	128 787 (63.8)	50 576 (63.8)	33 118 (64.6)	11 426 (64.2)	13 183 (63.8)	4897 (64.2)	219 560 (63.7)	78 490 (63.6)
Male	73 119 (36.2)	28 707 (36.2)	18 184 (35.4)	6379 (35.8)	7495 (36.2)	2734 (35.8)	124 933 (36.3)	44 941 (36.4)
Race and ethnicity, No. (%)								
Black	10 280 (5.1)	3851 (4.9)	3161 (6.2)	1024 (5.8)	1083 (5.2)	379 (5.0)	17 764 (5.2)	5973 (4.8)
White	180 414 (89.4)	70 885 (89.4)	43 305 (84.4)	15 171 (85.2)	18 072 (87.4)	6662 (87.3)	303 133 (88.0)	108 897 (88.2)
Other^a^	11 212 (5.6)	4547 (5.7)	4836 (9.4)	1610 (9.0)	1523 (7.4)	590 (7.7)	23 596 (6.8)	8561 (6.9)
Disabled	12 509 (6.2)	4088 (5.2)	4247 (8.3)	1282 (7.2)	1326 (6.4)	417 (5.5)	24 392 (7.1)	7562 (6.1)
Dual-eligible Medicare/Medicaid	17 072 (8.5)	5930 (7.5)	6131 (12.0)	2004 (11.3)	1972 (9.5)	658 (8.6)	34 715 (10.1)	11 360 (9.2)
Health services use (prior 12 mo)								
IRF	2157 (1.1)	854 (1.1)	745 (1.5)	280 (1.6)	285 (1.4)	101 (1.3)	4066 (1.2)	1561 (1.3)
SNF	8957 (4.4)	3165 (4.0)	2740 (5.3)	938 (5.3)	1038 (5.0)	380 (5.0)	16 293 (4.7)	5473 (4.4)
Hospital	56 137 (27.8)	21 171 (26.7)	14 886 (29.0)	5243 (29.4)	5541 (26.8)	2057 (27.0)	92 019 (26.7)	33 254 (26.9)

Abbreviations: IRF, inpatient rehabilitation facility; SNF, skilled nursing facility.

^a^ Includes American Indian and Alaska Native, Asian, Hispanic, and Hawaiian or other Pacific Islander) and other as classified from or reported in the Medicare claims data.

### Episode Spending Outcomes

**Figure 2.**
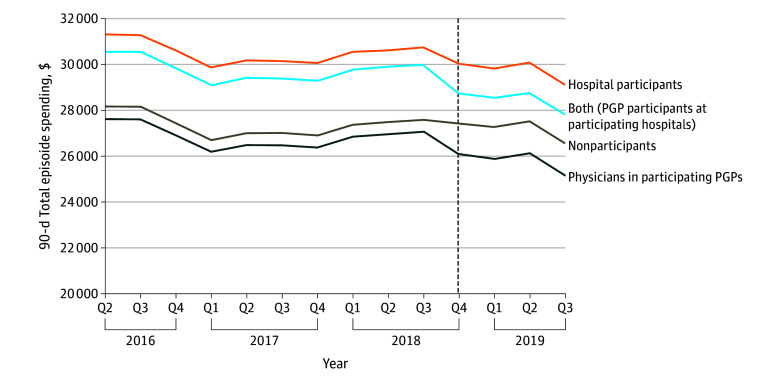
Adjusted Changes in 90-Day Total Joint Replacement Episode Spending, by BPCI-A−Participation From April 2016 to September 2019 Dashed line indicates inception of the Medicare Bundled Payments for Care Improvement Advanced (BPCI-A) model in October 2018; PGP, physician group practices; and Q, quarter.

Before BPCI-A began, unadjusted mean total episode spending at 90 days was $27 764 for nonparticipants, $26 483 for physicians in participating PGPs, and $29 854 for participating hospitals (eTable 3 in Supplement 1). At 90 days, receipt of joint replacement from physicians in participating PGPs was associated with an $855 differential reduction in spending compared to nonparticipants (adjusted difference in difference [aDID], −$855; 95% CI, −$1074 to −$636). Receipt of joint replacement from a participating hospital was associated with a $613 differential reduction (aDID, −$613; 95% CI, −$1039 to −$187). Beneficiaries who received surgery from both a BPCI-A−participating physician and participating hospital had a differential reduction of $1147 compared to those receiving care from nonparticipants (95% CI, −$1822 to −$472) (Table 2; Figure 2).

**Table 2.** Changes in Spending at 90 Days Between Bundled Payments for Care Improvement Advanced (BPCI-A) Participants vs Nonparticipants (NP), Before and After October 2018

Episode No. and spending	BPCI-A physician only		BPCI-A hospital only		BPCI-A both		NP		Adjusted difference-in-differences estimates			
	Before	After	Before	After	Before	After	Before	After	Physician vs NP	Hospital vs NP	Hospital vs physician	Both vs NP
Total joint replacement episodes, No.	201 906	79 283	51 302	17 805	20 678	7631	344 493	123 431	NA	NA	NA	NA
Total episode spending, unadjusted mean (SD), $	26 483 (16 248)	25 213 (15 080)	29 854 (18 929)	29 235 (18 334)	28 555 (17 357)	27 671 (16 523)	27 764 (17 211)	27 562 (17 101)	−855 (−1074 to −636)^a^	−613 (−1039 to −187)^b^	242 (−222 to 707)	−1147 (−1822 to −472)^b^

Abbreviation: NA, not applicable.

^a^ *P* < .001.

^b^ *P* < .01.

At 30 days, there were statistically significant differential reductions in spending associated with receiving care from participating physicians (aDID, −$785; 95% CI, −$975 to −$596), hospitals (aDID, −$648; 95% CI, −$1023 to −$273), and both participating physicians and hospitals (aDID, −$1007; 95% CI, −$1607 to −$407; eTables 5 and 6 in Supplement 1).

### Quality Outcomes

No significant differential changes in mortality, hospital readmissions, ED visits, or joint replacement complications were observed for patients treated by either BPCI-A participant type compared to nonparticipants at either 30 or 90 days. eTables 4 and 6 in Supplement 1 provide additional details.

### Utilization Outcomes

Joint replacement by physicians in BPCI-A−participating PGPs was associated with differentially lower rates of discharge to inpatient rehabilitation facility (IRF) (aDID, −1.3 percentage points [pp]; 95% CI, −1.7 to −0.9 pp), discharge to SNF (aDID, −1.8 pp; 95% CI, −2.6 to −1.0 pp), and SNF length of stay (aDID, −0.8 days; 95% CI, −1.2 to −0.3 days) at 90 days compared to nonparticipants. These beneficiaries had significantly fewer days enrolled in home health (aDID, −0.8 days; 95% CI, −1.0 to −0.6 days) and a differential increase in outpatient physician visits within 7 days of discharge from the index admission (aDID, 2.9 pp; 95% CI, 2.0 to 3.8 pp) (Table 3).

**Table 3.** Changes in Utilization at 90 Days Between Bundled Payments for Care Improvement Advanced (BPCI-A) Participants vs Nonparticipants (NP), Before and After October 2018

Unadjusted outcome	Beneficiaries, No. (%)											
	BPCI-A physician only		BPCI-A hospital only		BPCI-A both		NP		Adjusted difference-in-differences estimates			
	Before	After	Before	After	Before	After	Before	After	Physician vs NP	Hospital vs NP	Hospital vs physican	Both vs NP
Discharge to IRF	7686 (3.8)	2238 (2.8)	3708 (7.2)	1082 (6.1)	1136 (5.5)	356 (4.7)	16 171 (4.7)	6222 (5.0)	−0.013 (−0.017 to −0.009)^a^	−0.014 (−0.022 to −0.006)^b^	−0.001 (−0.010 to 0.007)	−0.014 (−0.027 to −0.010)^c^
Discharge to SNF	51 184 (25.4)	16 439 (20.7)	15 434 (30.1)	4974 (27.9)	6285 (30.4)	2090 (27.4)	94 881 (27.5)	31 119 (25.2)	−0.018 (−0.026 to −0.010)^a^	−0.008 (−0.025 to 0.010)	0.010 (−0.008 to 0.028)	−0.028 (−0.055 to −0.010)^c^
Discharge to home health	58 762 (29.1)	21 468 (27.1)	16 625 (32.4)	6067 (34.1)	7856 (38.0)	2090 (27.4)	113 873 (33.1)	39 602 (32.1)	−0.009 (−0.022 to 0.003)	0.028 (0.007 to 0.050)^b^	0.038 (0.015 to 0.060)^b^	0.015 (−0.028 to 0.057)
SNF length of stay, mean (SD), d	23.0 (20.1)	23.2 (19.7)	25.3 (20.8)	25.4 (20.8)	22.7 (19.7)	22.0 (19.2)	24.8 (24.2)	25.3 (21.2)	−0.76 (−1.21 to −0.31)^b^	−0.72 (−1.39 to −0.05)^c^	0.04 (−0.67 to 0.76)	−1.32 (−2.26 to −0.38)^b^
HHA, mean (SD), d	11.5 (7.8)	10.8 (7.7)	12.8 (8.3)	12.4 (8.1)	11.7 (8.0)	10.6 (7.6)	12.0 (7.8)	11.9 (7.8)	−0.80 (−1.01 to −0.58)^a^	−0.03 (−0.58 to 0.01)	0.51 (0.15 to 0.87)^b^	−0.99 (−1.48 to −0.51)^a^
7-d Postdischarge outpatient physician visits	34 522 (17.1)	16 848 (21.3)	4042 (7.9)	1375 (7.7)	1901 (9.2)	697 (9.1)	39789 (11.6)	15136 (12.3)	0.029 (0.020 to 0.038)^a^	−0.005 (−0.014 to 0.005)	−0.034 (−0.045 to −0.022)^a^	0.006 (−0.014 to 0.026)

Abbreviations: HHA, home health aide; IRF, inpatient rehabilitation facility; SNF, skilled nursing facility.

^a^ *P* < .001.

^b^ *P* < .01.

^c^ *P* < .05.

In comparison, beneficiaries who received joint replacement from BPCI-A−participating hospitals also had differentially lower rates of discharge to IRF (aDID, −1.4 pp; 95% CI, −2.2 to −0.6 pp) and SNF length of stay (aDID, −0.7 days; 95% CI, −1.4 to −0.5 days). However, these beneficiaries had a differential increase in discharge to home health (aDID, 2.8 pp; 95% CI, 0.7 to 5.0 pp). There was no significant difference in discharge to SNF and no significant change in 7-day postdischarge outpatient physician visits for beneficiaries at participating hospitals. For both hospitals and physicians, 30-day utilization outcomes were directionally consistent with 90-day outcomes (eTable 6 in Supplement 1).

### Sensitivity and Exploratory Analyses

Findings for the primary outcome were robust and unchanged with sensitivity analyses (eTables 7-12 in Supplement 1). In exploratory analyses, physicians in participating PGPs demonstrated a statistically significant differential increase in share of beneficiaries with prior SNF use (0.2%; 95% CI, 0% to 0.4%) and differential decrease in share of beneficiaries with prior hospital use (−1.5%; 95% CI, −1.9% to −1.1%) compared to nonparticipants (eTable 13 in Supplement 1). Participating hospitals demonstrated no significant changes in these shares compared to nonparticipants, and there were no significant differential changes in other beneficiary characteristics. In the exploratory analysis of spending during 31 to 90 days, we observed an unadjusted increase in mean total episode spending ($53) and spending on readmissions ($63) among hospital participants, while physician participants demonstrated decreased mean total episode spending (−$149) and spending on readmissions (−$69) (eTable 14 in Supplement 1).

## Discussion

In this cohort study, treatment by physicians in PGPs that participated in a Medicare bundled-payment model was associated with differentially lower episode spending than treatment by nonparticipants. Hospital participation was also associated with savings. The reduction in spending associated with hospital participation is consistent with findings from prior bundled-payment models.^8,20,29,30,31^ Our analysis adds to prior evidence with what is to our knowledge the first independent and peer-reviewed evaluation to report on savings associated with treatment by BPCI-A−participating PGPs. Given that PGPs outpaced hospitals in joint replacement episode volume, these findings have important implications for the design of TEAM—to engage physicians in this forthcoming mandatory bundled-payment model that will allow only hospital participation.

This study made 3 advances from prior evaluations of BPCI-A. First, in addition to episodes for participating hospitals, we included episodes for physicians in participating PGPs. PGPs are responsible for 73% of joint replacement episodes among BPCI-A participants, yet prior studies likely (incorrectly) assigned these episodes to the hospitals where they were performed.^4,7,15^ Second, this study focused on a single surgical procedure with high national uptake in this bundled-payment model, rather than aggregating multiple surgical and medical conditions. Performance in BPCI-A may vary considerably across the 32 clinical conditions in the model, highlighting the need to measure changes in spending and utilization separately by clinical episode type.^6,15^ Third, because decisions to participate are made at the service-line level, we retained BPCI-A participants for nonorthopedic conditions in the comparison group, which more accurately reflects program rules.

Our study findings have 3 key implications. First, cost savings among beneficiaries treated by physicians in BPCI-A−participating PGPs suggest that future bundled-payment models should engage physician group practices. Our difference-in-differences estimates translate to 3.1% savings for patients treated by physician participants, 2.2% for hospital participants, and 4.1% for both, with the hospital estimates consistent with findings from prior bundled-payment models.^8,20,29,30,31^ Reductions in spending attributable to physicians in participating PGPs accounted for 78% of the total savings in our study, reflected by the 73% share of PGP episodes. In future bundled-payment models, approaches to involving PGPs may include gainsharing to transfer a portion of the savings to physicians or additional incentives for coordination or coparticipation, or allowing direct participation.^23,32,33,34^ Gainsharing approaches have demonstrated success in prior episode-based payment models for orthopedic procedures, particularly those that are surgeon led.^9,10,35^ The potential for synergy is supported by the finding that beneficiaries treated by both participating physician and hospitals demonstrated the greatest magnitude of savings.

Second, the different mechanisms by which physicians and hospitals appeared to change postacute care utilization highlight their relative strengths in influencing care after discharge.^36^ Savings coming from postacute care utilization are consistent with findings from prior bundled-payment programs.^4,5,6,21,31^ BPCI-A participation was associated with a differential increase in home health utilization among participating hospitals but a decrease among participating physicians. In addition, differentially greater postdischarge outpatient physician visits among beneficiaries treated by physicians but not by hospitals suggest that physicians may partially realize savings through their ability to coordinate outpatient follow-up for patients. Future models that focus only on hospitals may seek to encourage hospitals to adopt strategies for which physicians may inherently be more adept, such as scheduling timely outpatient follow-up care. The different approaches adopted by PGPs compared with hospitals underscore the importance of patient-reported outcome measures to monitor quality of the patient experience, which will be a feature in TEAM.^19,37,38^

Third, differentially lower spending observed at 30 days suggests that it may be feasible to shorten episode duration, as proposed in TEAM, without compromising savings. Most spending reductions occurred within the first month of surgery, potentially because hospitals and physicians have limited capability to influence outcomes and utilization distant from the index procedure.^39^ Decreasing episode duration with emphasis on transferring accountability to outpatient primary and specialty care may better align financial responsibility with actual oversight for patient outcomes, especially for hospital participants.^40^ Allowing overlap between episode-based bundled payments and population-based health services may further enhance synergy between hospitals and physicians accountable for patient outcomes.^41,42,43^

Notably, we must consider that favorable patient selection may be responsible for a portion of the spending and utilization results observed for physicians.^31^ Patients treated by physicians in participating PGPs appeared healthier on average in both the pre- and postperiods, although we used methods to account for these differences. These healthier patients may have less need for postacute care, enabling PGPs to be more responsive to incentives to reduce it. We also identified a reduction in the share of patients with prior hospital use over time associated with participating physicians but not participating hospitals, although this was offset by an increase in the share with past SNF use. While not causal, these exploratory findings may reflect dynamics in which bundled-payment participants may select against patients who may be costly or complex to care for, beyond what is captured through risk adjustment.^44,45^ It is important for policymakers to monitor for favorable patient selection, which may exacerbate long-standing disparities in access to joint replacement for historically marginalized groups.^46,47,48,49,50,51,52^ Approaches to mitigate this may relate to accounting for social factors in risk adjustment.^53,54^ While it is possible that mandatory models may protect against some selection effects, there is mixed evidence of their overall impact on disparities.^55,56,57,58^

### Limitations

This study has limitations. First, the study focused on the first 2 years of the BPCI-A model to avoid the associated effects of COVID-19 changes as well as exit and entry during subsequent model years; this approach may affect generalizability of the findings to later years of BPCI-A as well as to other programs. Second, BPCI-A is a voluntary model, increasing the potential for residual unobserved differences between matched groups. However, evidence from 2 prior joint replacement bundled-payment models suggests that voluntary and mandatory participation did not meaningfully affect program results.^59^ Third, this study excluded outpatient joint replacement because it was not part of BPCI-A during our study period, although it has become more common and was included in subsequent model years. Fourth, participation in BPCI-A is at the PGP level but there is no physician group identifier for nonparticipating physicians, so we matched at the physician level and included only hospital-level fixed effects. If physicians participated in multiple groups, we would not know whether the episode was under the participating PGP or not, although this would bias our results toward the null. Fifth, we were unable to identify and exclude physicians in PGPs who initially elected to participate in BPCI-A but withdrew in March 2019, although the inclusion of these physicians in the comparison group would also bias our results toward the null. Finally, our study was not designed or intended to address broader questions in bundled-payment policy, such as which conditions to include, how treatment effects vary across model years and design changes, and how gross and net savings differ. Instead, we took a focused approach to evaluate PGP participation in joint replacement given its salience to the current policy surrounding TEAM.

## Conclusions

The findings of this cohort study indicate that both physician and hospital BPCI-A participation for joint replacement procedures were associated with reduced health care spending. These findings highlight the importance of encouraging alignment between physicians and hospitals in the design of future models as well as continued opportunities to improve cost-efficiency and quality through bundled payments.

## Notes

Supplement 1. eMethods 1. Detailed claim-level and episode-level inclusion and exclusion criteria. eMethods 2. Identification of participating physicians. eMethods 3. Orthopedic service line grouping. eMethods 4. Cost standardization. eMethods 5. BPCI Advanced program details. eMethods 6. Matching strategy and associated variables. eMethods 7. Model specification. eFigure. Parallel trends plots. eTable 1. Characteristics of BPCI Advanced and Non-BPCI Advanced Hospitals, Before and After Propensity Score Matching eTable 2. Characteristics of Physicians in BPCI Advanced and Non-BPCI Advanced PGPs, Before and After Propensity Score Matching eTable 3. Unadjusted Changes in 90-day Outcomes, Baseline versus Intervention Period eTable 4. Difference-in-Differences Estimates for Changes in 90-day Outcomes Associated With Bundled Payments eTable 5. Unadjusted Changes in 30-day Outcomes, Baseline versus Intervention Period eTable 6. Difference-in-Differences Estimates for Changes in 30-day Outcomes Associated With Bundled Payments eTable 7. Sensitivity Analysis, 1:1 Matching eTable 8. Sensitivity Analysis, Treating “Both” Group as PGP eTable 9. Sensitivity Analysis, Overall Program Effect eTable 10. Sensitivity Analysis, Washout Period eTable 11. Sensitivity Analysis, Separate Participant Effects eTable 12. Sensitivity Analysis, No Prior BPCI Advanced Participation eTable 13. Exploratory Analyses, Differential Changes in Patient Characteristics eTable 14. Exploratory Analyses, Unadjusted Changes in 31-90 Day Outcomes

Supplement 2. Data Sharing Statement
