# Supplementary material for: Physician and Hospital Performance in Medicare’s Updated Bundled-Payment Model for Joint Replacement
Source: JAMA Health Forum. 2025 Jul 25;6(7):e251930. doi: 10.1001/jamahealthforum.2025.1930 (PMC12767874; doi:10.1001/jamahealthforum.2025.1930)
Supplement: Supplement 1. — eMethods 1. Detailed claim-level and episode-level inclusion and exclusion criteria. eMethods 2. Identification of participating physicians. eMethods 3. Orthopedic service line grouping. eMethods 4. Cost standardization. eMethods 5. BPCI Advanced program details. eMethods 6. Matching strategy and associated variables. eMethods 7. Model specification. eFigure. Parallel trends plots. eTable 1. Characteristics of BPCI Advanced and Non-BPCI Advanced Hospitals, Before and After Propensity Score Matching eTable 2. Characteristics of Physicians in BPCI Advanced and Non-BPCI Advanced PGPs, Before and After Propensity Score Matching eTable 3. Unadjusted Changes in 90-day Outcomes, Baseline versus Intervention Period eTable 4. Difference-in-Differences Estimates for Changes in 90-day Outcomes Associated With Bundled Payments eTable 5. Unadjusted Changes in 30-day Outcomes, Baseline versus Intervention Period eTable 6. Difference-in-Differences Estimates for Changes in 30-day Outcomes Associated With Bundled Payments eTable 7. Sensitivity Analysis, 1:1 Matching eTable 8. Sensitivity Analysis, Treating “Both” Group as PGP eTable 9. Sensitivity Analysis, Overall Program Effect eTable 10. Sensitivity Analysis, Washout Period eTable 11. Sensitivity Analysis, Separate Participant Effects eTable 12. Sensitivity Analysis, No Prior BPCI Advanced Participation eTable 13. Exploratory Analyses, Differential Changes in Patient Characteristics eTable 14. Exploratory Analyses, Unadjusted Changes in 31-90 Day Outcomes [file jamahealthforum-e251930-s001.pdf]

# Supplemental Online Content

Crowley AP, Kilaru AS, Huang QE, et al. Physician and hospital performance in Medicare's updated bundled-payment model for joint replacement. *JAMA Health Forum.* Published online July 25, 2025. doi:10.1001/jamahealthforum.2025.1930

**eMethods 1.** Detailed claim-level and episode-level inclusion and exclusion criteria.

**eMethods 2.** Identification of participating physicians.

**eMethods 3.** Orthopedic service line grouping.

**eMethods 4.** Cost standardization.

**eMethods 5.** BPCI Advanced program details.

**eMethods 6.** Matching strategy and associated variables.

**eMethods 7.** Model specification.

**eFigure.** Parallel trends plots.

**eTable 1.** Characteristics of BPCI Advanced and Non-BPCI Advanced Hospitals, Before and After Propensity Score Matching

**eTable 2.** Characteristics of Physicians in BPCI Advanced and Non-BPCI Advanced PGPs, Before and After Propensity Score Matching

**eTable 3.** Unadjusted Changes in 90-day Outcomes, Baseline versus Intervention Period

**eTable 4.** Difference-in-Differences Estimates for Changes in 90-day Outcomes Associated With Bundled Payments

**eTable 5.** Unadjusted Changes in 30-day Outcomes, Baseline versus Intervention Period

**eTable 6.** Difference-in-Differences Estimates for Changes in 30-day Outcomes Associated With Bundled Payments

**eTable 7.** Sensitivity Analysis, 1:1 Matching

**eTable 8.** Sensitivity Analysis, Treating "Both" Group as PGP

**eTable 9.** Sensitivity Analysis, Overall Program Effect

**eTable 10.** Sensitivity Analysis, Washout Period

**eTable 11.** Sensitivity Analysis, Separate Participant Effects

© 2025 Crowley AP et al. *JAMA Health Forum.*

**eTable 12.** Sensitivity Analysis, No Prior BPCI Advanced Participation

**eTable 13.** Exploratory Analyses, Differential Changes in Patient Characteristics

**eTable 14.** Exploratory Analyses, Unadjusted Changes in 31-90 Day Outcomes

This supplemental material has been provided by the authors to give readers additional information about their work.

© 2025 Crowley AP et al. *JAMA Health Forum.*

**eMethods 1. Detailed claim-level and episode-level inclusion and exclusion criteria.**

1) **Claim-level (following BPCI Advanced Model Years 1 and 2 exclusion rules):**
   i) Identify all index admissions with DRG 469 or 470.
   ii) Add all claims associated with the index admission.
   iii) Apply a 90-day non-overlapping rule by assigning overlapping episodes (for joint replacement or any other BPCI Advanced condition) to the earlier hospitalization
   iv) Add all inpatient, professional, outpatient, institutional post-acute care (PAC, includes inpatient rehabilitation facility (IRF), skilled nursing facility (SNF), and long-term care (LTC)), home health, hospice, and durable medical equipment claims within 90 days from the index procedure.
   v) Exclude claims if the Medicare payment amount is negative or 0.
   vi) Exclude certain readmissions based on BPCI-A MY1/2 exclusion list (transplants, trauma, and cancer).
   vii) Exclude IPPS hospital claims with new technology add-ons.
   viii) Exclude Part B claims for drugs on the average sales price.
   ix) Exclude Part B claims with blood clotting factor.
   x) Exclude OPPS outpatient claims with pass-through payment for medical devices.
   xi) Exclude claims that represent per-bene-per-month payments from Carrier and Hospice claims.
2) **Episode-level**
   i) Exclude if beneficiary is not continuously enrolled in Medicare Parts A and B during the episode period and a 6-month lookback period.
   ii) Exclude if beneficiary is eligible for Medicare due to ESRD during the episode period or a 90-day lookback period.
   iii) Exclude if beneficiary is enrolled in Medicare Advantage during the episode period or a 6-month lookback period.
   iv) Exclude if beneficiary has primary payer other than Medicare during the episode period or a 6-month lookback period.
   v) Exclude if beneficiary dies during index admission.

© 2025 Crowley AP et al. *JAMA Health Forum.*

**eMethods 2. Identification of participating physicians.**

We identified physicians participating in BPCI Advanced via physician group practices (PGPs) by first linking two public files: the BPCI Advanced Data Crosswalk and the BPCI Advanced Participant List. The crosswalk file contains aggregated information for reconciliated BPCI Advanced index hospitalizations. Information includes Bundled Payment ID (BPID, a unique participant identifier), performance period, hospital provider number of the index admission, attending physician NPIand operating physician NPI. The participant list includes BPID, organization legal name, and participant type (PGP or hospital). The following steps were taken to identify episodes attributed to physicians who participated in BPCI Advanced:

1. Linked hospitalizations in the BPCI Advanced Data Crosswalk file to the BPCI Advanced Participant List on BPID to identify records from the crosswalk file that are attributed to PGPs participating in lower extremity joint replacement.
2. Merged the updated crosswalk file with Medicare inpatient claims to identify BPCI Advanced index admissions based on presence of matching hospital where the procedure was performed, attending and/or operating NPI, and performance period.
3. Removed matched inpatient claims (index admissions) if corresponding Part B physician claims were not found.
4. Since participation takes place at the PGP level, it is not known whether the attending or operating physician associated with the index admission was the physician in a participating physician group. To perform physician matching, we used the following logic to attribute the index admission to one physician:
    a. If the attending was the same as the operating physician, we assigned the attending physician as the participating one; if not, we used the operating physician.

**eMethods 3. Orthopedic service line grouping.**

We used Model Year 4 (2020) definitions of service line groups. The orthopedic service line group contains:
1) Hip and femur procedures
2) Lower extremity and humerus procedures
3) Major joint replacement of the lower extremity
4) Major joint replacement of the upper extremity
5) Double joint replacement of the lower extremity
6) Fractures femur and hip/pelvis.

If non-joint replacement BPCI Advanced participants ever participated in any of these conditions, they were removed from the control group. This means that BPCI Advanced participants who may have participated in other conditions for BPCI Advanced were retained in the control group, provided that none of those conditions were in the orthopedic service line group.

© 2025 Crowley AP et al. *JAMA Health Forum.*

**eMethods 4. Cost standardization.**
Medicare payments are necessarily adjusted to account for geographic factors and provider characteristics. In order to compare relative levels of spending, we assigned standardized costs for claims following prior methods.[1] This method assigns the same standardized cost for a particular service regardless of the specific characteristics of the patient and provider involved, allowing for a comparison in spending across providers. Costs were standardized to 2019 dollars.

**Inpatient Payments**
To standardize inpatient costs, we multiplied the corresponding Diagnosis-Related Group (DRG) weight by the 2019 national standard base payment. DRGs are a classification system used to categorize inpatient stays for the purpose of reimbursement, and weights reflect the relative resource intensity of the inpatient stay. Documentation of the 2019 national standard base payment ($6,105.49) and DRG weights and were downloaded from CMS.gov.[2]

**Health Care Provider Payments**
Health care provider payments (claim line file for Part B Carrier and Durable Medical Equipment) were defined as payments for physician services. To standardize costs, we used the 2010-2019 national Medicare fee schedules to assign a standardized cost based on the Healthcare Common Procedure Coding System (HCPCS) code.[3] For HCPCS codes that were not used in 2019, we converted the standardized cost to 2019 dollars by multiplying by the inflation rate. Claim lines with a place-of-service code indicating a facility were assigned the facility fee and claim lines with other place-of-service codes were assigned the non-facility fee. The unit cost was multiplied by the number of units and assigned at the claim line level. Claim level standardized costs were obtained by summing corresponding line level costs. For Level II HCPCS codes identifying produces, supplies, and services not under the national fee schedule, we assigned a standardized cost based on the mean payment by year, converting to 2019 dollars.

---

[1] Tsai TC, Joynt KE, Wild RC, Orav EJ, Jha AK. Medicare's Bundled Payment initiative: most hospitals are focused on a few high-volume conditions. *Health Aff (Millwood)*. 2015;34(3):371-380. doi:10.1377/hlthaff.2014.0900

[2] CMS. 2019 Inpatient Prospective Payment System Final Rule (Table 1ABC: base payment information, Table 5: DRG weights.) Available at: https://www.cms.gov/medicaremedicare-fee-service-paymentacuteinpatientppsacute-inpatient-files-download/files-fy-2019-final-rule-and-correction-notice. Accessed November 23, 2024.

[3] CMS. 2019 Physician Fee Schedule. Available at: https://www.cms.gov/Medicare/Medicare-Fee-for-Service-Payment/PhysicianFeeSched/PFS-National-Payment-Amount-File. Accessed November 23, 2024.

© 2025 Crowley AP et al. *JAMA Health Forum.*

**Outpatient Payments**

Outpatient payments (Outpatient Revenue Center File) were defined as payments for outpatient services, generally performed at emergency departments and outpatient hospital clinics. To standardize costs, we used the 2014-2019 Ambulatory Payment Classification (APC) payment from CMS for claim lines under OPPS. For APC that were not used in 2019, we converted the standardized cost to 2019 dollars by multiply the inflation rate. For claim lines not under the Outpatient Prospective Payment System (OPPS), we used the corresponding HCPCS code mean payment by year, converted to 2019 dollars, and adjusted for the local Medicare Wage Index which was assumed to be 1. APCs are a classification system analogous to DRGs for inpatient payments, and weights reflect the relative resource intensity of the service. Documentation of the APC payments were downloaded from CMS.gov.[4] The 2019 national standard base payment (conversion factor) is $79.546 (page 45 in final rule).

**Post-Acute Care Payments**

Post-acute care payments were defined as payments for skilled nursing facilities (SNF), inpatient rehabilitation facilities (IRF), and home health agencies (HHA). To standardize SNF payments (SNF Revenue Center File), we used the 2019 national Resource Utilization Group (RUG) rate schedule. Unit costs were multiplied by the revenue center unit count and assigned at the claim line level. Claim level standardized costs were obtained by summing corresponding line level costs. RUG categories reflect the relative resource needs of the patient, indicated by the HCPCS code. For SNF claims with missing RUG, we used the actual Medicare payment amount. Documentation of the 2019 national RUG rate schedule was downloaded from CMS.gov.[5]

To standardize IRF payments (Inpatient Claim file), we multiplied the corresponding Diagnosis-Related Group (DRG) weight by the 2019 IRF national standard base payment ($16,021). IRF claims are indicated by the CMS Hospital Provider Number. Documentation of the 2019 national standard base payment and DRG weights were downloaded from CMS.gov.[6]

For HHA payments (HHA Revenue Center File), the mean value of the revenue center payment by year was calculated for each Home Health Resource Group (HHRG). HHRG categories reflect the relative resource needs of the patient, indicated by the first four digits of the Health Insurance Prospective Payment (HIPPS) code. We multiplied the HHRG weight by 2019 HHA base

---

[4] CMS. 2019 Outpatient Prospective Payment System Policy Files. Available at: https://www.cms.gov/Medicare/Medicare-Fee-for-Service-Payment/HospitalOutpatientPPS/Annual-Policy-Files-Items/2019-Annual-Policy-Files. Accessed November 23, 2024.

[5] CMS. 2019 Skilled Nursing Facility Prospective Payment System. Available at: https://www.cms.gov/medicare/payment/prospective-payment-systems/skilled-nursing-facility-snf/list-federal-regulations. Accessed November 23, 2024.

[6] CMS. 2019 Inpatient Rehabilitation Facility Final Rule. Available at: https://www.govinfo.gov/content/pkg/FR-2018-08-06/pdf/2018-16517.pdf. Accessed November 23, 2024.

© 2025 Crowley AP et al. *JAMA Health Forum.*

payment ($3154.27) to get standardized revenue center payment (line level).[7] Claim level standardized costs were obtained by summing corresponding line level costs.

**eMethods 5. BPCI Advanced program details.**
Physician group practices and hospitals could elect to participate in BPCI Advanced beginning in October 2018. Model Years 1 and 2 of BPCI Advanced (October 2018 – December 2019) allowed hospitals or physician groups to bear financial responsibility for any of 32 distinct clinical episodes. BPCI Advanced includes all hospital and physician services from the anchor stay or procedure as well as post-acute services, including home and hospice care. It builds on BPCI by lengthening all episodes to 90 days, putting up to 10% of payments at risk with a novel quality component, and risk adjusting payments based on hospital and patient characteristics and historical costs. It also streamlines four separate risk tracks into one orogram. Unlike in BPCI, PGP participants in BPCI Advanced always take precedence over hospitals.

**eMethods 6. Matching strategy and associated variables.**
We matched hospitals according to hospital-level variables (ownership, urban status, teaching status, resident-to-bed ratio, % Medicare days, bed count, health system affiliation, disproportionate share status), joint replacement-specific variables (joint replacement volume, proportions of discharges to highest-volume SNF, and hospital market share), and market-level variables at the hospital referral region (HRR) level (population size, % of Medicare beneficiaries from low-income ZIP codes, Medicare Advantage penetration, SNF beds per 10,000 patients, and hospital concentration defined by Herfindahl-Hirschman Index (HHI)). We performed an exact match on teaching status. We followed prior literature in selecting which variables to match upon and the matching procedure itself.[8]

We matched physicians at the NPI level using physician-level variables (age, sex, annual service volume, annual payments, number of beneficiaries cared for, proportion of services provided inpatient, core-based statistical area (CBSA) type (metropolitan, micropolitan, or rural), health system affiliation, care for patients at a teaching hospital, and proportion of

---

[7] CMS. 2019 Home Health Prospective Payment System. Available at: https://www.cms.gov/Medicare/Medicare-Fee-for-Service-Payment/HomeHealthPPS/Home-Health-Prospective-Payment-System-Regulations-and-Notices. Accessed November 23, 2024.
[8] Liao JM, Huang Q, Wang E, Linn K, Shirk T, Zhu J, Cousins D, Navathe AS. Performance of Physician Groups and Hospitals Participating in Bundled Payments Among Medicare Beneficiaries. *JAMA Health Forum*. 2022 Dec 2;3(12):e224889.

© 2025 Crowley AP et al. *JAMA Health Forum.*

admissions from each of dual-eligible, Black, joint replacement, and high-volume hospital), practice-level variables (number of providers, multi-specialty practice status), and the same market-level variables as above.

**eMethods 7. Model specification.**

We estimated primary and secondary outcomes using a generalized linear model with quarter-year, hospital, and DRG fixed effects.

$$
\begin{aligned}
Y_{\text{!,\#(\%),',(}} = {} & BPCI\_PGP\_ever_{\text{!}} + BPCI\_Hosp\_ever_{\text{!}} + POST_{\text{\#/\%}} + BPCI\_PGP\_ever_{\text{!}} * POST_{\text{\#/\%}} + BPCI\_Hosp\_ever_{\text{!}} * POST_{\text{\#/\%}} \\
& + BPCI\_PGP\_ever_{\text{!}} * BPCI\_Hosp\_ever_{\text{!}} + BPCI_{\text{*+,-!"!\#}_{\text{!}}} * BPCI_{\text{-.-!"!\#}_{\text{!}}} * POST_{\text{\#/\%}} + T_{\text{'}} + HOSP + DRG + Covar \\
& + MA\_Pene_{\text{(}} * T_{\text{'}} + ACOPene_{\text{(}} * T_{\text{'}} + HHI_{\text{\#}} * T_{\text{'}} + ElixhauserScore_{\text{!}} * T_{\text{'}}
\end{aligned}
$$

for patient *i*, hospital *h*, physician *p* in PGP, quarter *q*, market (region) *r*.

© 2025 Crowley AP et al. *JAMA Health Forum.*

**eFigure. Parallel trends plots.**

Plots demonstrate fully adjusted outcomes for each of PGP- and hospital-initiated episodes during the pre-period. Estimates are in reference to 2018 Q4 when BPCI Advanced began. All plots suggest that parallel trends assumption holds for primary outcome of 90-day total episode spending as well as secondary outcomes including 30-day total episode spending, 90-day mortality, and 90-day readmissions.

**Outcome: 90-day standardized total spending.**

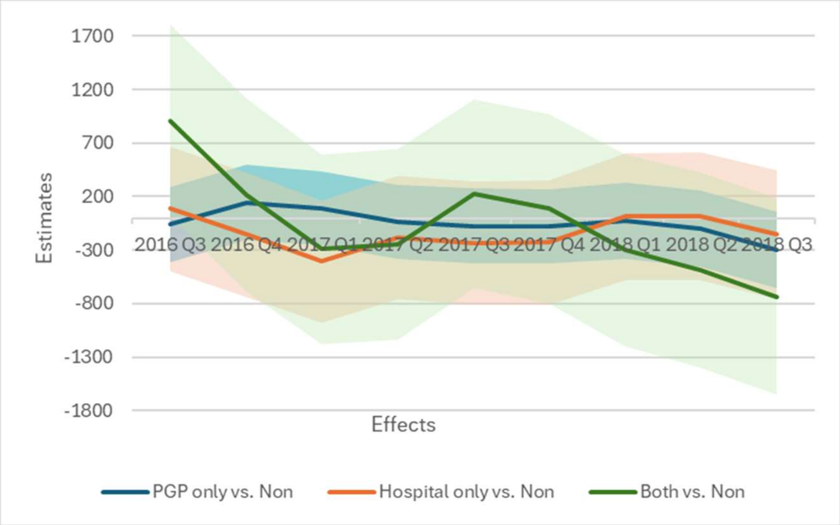


**Outcome: 30-day standardized total spending.**

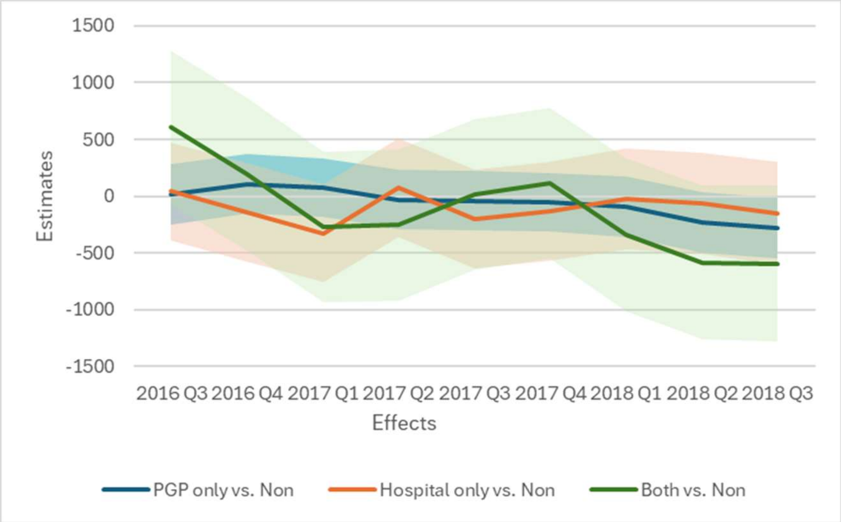


© 2025 Crowley AP et al. *JAMA Health Forum.*

**Outcome: 90-day readmissions.**

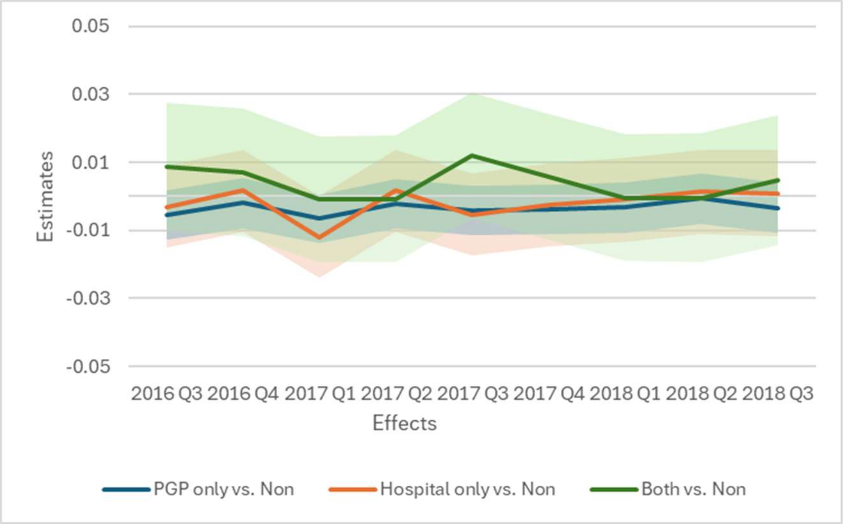


**Outcome: 90-day mortality.**

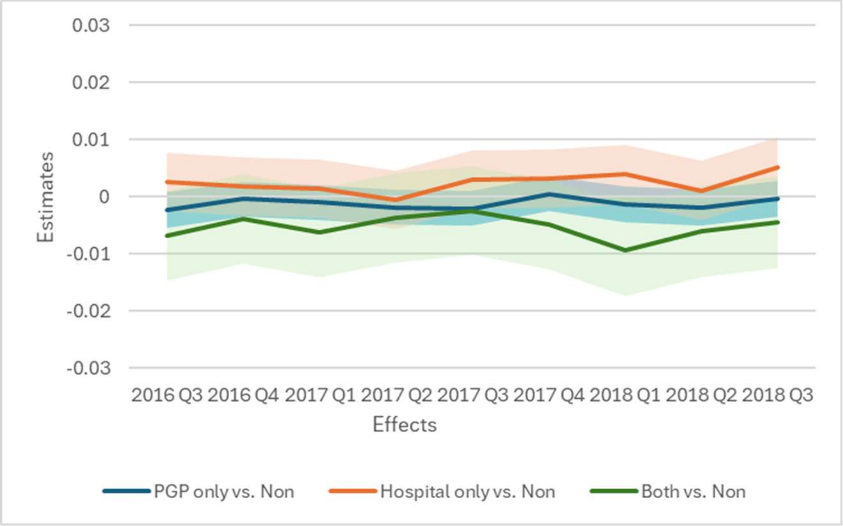


© 2025 Crowley AP et al. *JAMA Health Forum.*

**eTable 1. Characteristics of BPCI Advanced and Non-BPCI Advanced Hospitals, Before and After Propensity Score Matching.**

Hospitals that had ever (BPCI-A ever) or never (BPCI-A never) participated in BPCI Advanced were matched according to the 3:1 propensity score matching procedure detailed above in eMethods 6. Standardized mean differences (SMD) within +/- 0.1 suggest that our matching procedure performed well.

| | Before matching | | | After matching | | | |
|---|---|---|---|---|---|---|---|
| | BPCI-A never | BPCI-A ever | SMD | BPCI-A never | | BPCI-A ever | SMD |
| **Total no. of Hospitals** | 2347 | 177 | | 432 | | 174 | |
| | | | | | | | |
| **Hospital characteristics** | | | | **Unweighted** | **Weighted** | | |
| Ownership N (%) | | | 0.3316 | | | | 0.0527 |
| Non-profit | 1526 (65.4) | 107 (60.5) | | 275 (63.7) | 109 (62.8) | 107 (61.5) | |
| For-profit | 508 (21.8) | 61 (34.5) | | 134 (31.0) | 56 (32.0) | 58 (33.3) | |
| Government | 298 (12.8) | 9 (5.1) | | 23 (5.3) | 9 (5.2) | 9 (5.2) | |
| Urban status N (%) | | | 0.1381 | | | | -0.0717 |
| Urban | 2220 (95.2) | 173 (97.7) | | 425 (98.4) | 172 (98.7) | 170 (97.7) | |
| Rural | 112 (4.8) | 4 (2.3) | | 7 (1.6) | 2 (1.3) | 4 (2.3) | |
| Teaching hospital status N (%) | | | 0.2597 | | | | 0 |
| Major teaching | 172 (7.4) | 19 (10.7) | | 41 (9.5) | 18 (10.3) | 18 (10.3) | |
| Minor teaching | 1100 (47.2) | 97 (54.8) | | 231 (53.5) | 97 (55.7) | 97 (55.7) | |
| Non-teaching | 1060 (45.5) | 61 (34.5) | | 160 (37.0) | 59 (33.9) | 59 (33.9) | |
| Residents to beds ratio, mean (SD) | 0.1 (0.2) | 0.1 (0.3) | 0.1133 | 0.1 (0.3) | 0.1 (0.2) | 0.1 (0.3) | -0.0309 |
| Proportion of Medicare days*, mean (SD) | 51.2 (13.1) | 51.3 (11.0) | 0.002 | 51.9 (11.9) | 51.6 (7.4) | 51.3 (11.0) | -0.0246 |

© 2025 Crowley AP et al. *JAMA Health Forum.*

| | | | | | | | |
|---|---|---|---|---|---|---|---|
| Total hospital bed count, mean (SD) | 224.0 (212.6) | 288.4 (249.0) | 0.2782 | 266.7 (225.7) | 287.8 (154.2) | 290.8 (249.5) | 0.0146 |
| Health system affiliated, N (%) | 1680 (72.0) | 151 (85.3) | 0.3283 | 360 (83.3) | 149 (85.4) | 150 (86.2) | 0.022 |
| Disproportionate share payment, mean (SD) | 381769.4 (667178.1) | 560060.2 (764731.3) | 0.2484 | 500419.7 (683742.6) | 552140.7 (467881.2) | 563540.9 (767975.8) | 0.0179 |
| | | | | | | | |
| **Hospital utilization** | | | | **Unweighted** | **Weighted** | | |
| Lower extremity joint replacement Medicare annual admissions, mean (SD) | 1743.7 (1539.1) | 1870.9 (1576.6) | 0.0816 | 1835.6 (1435.6) | 1900.6 (913.6) | 1891.3 (1580.6) | -0.0072 |
| Proportion of discharges to highest volume SNF, mean (SD) | 32.6 (18.5) | 26.1 (14.4) | -0.3887 | 26.8 (14.1) | 26.0 (9.0) | 26.2 (14.4) | 0.0156 |
| Hospital market share, mean (SD) | 9.9 (14.9) | 11.7 (13.8) | 0.1207 | 11.4 (15.5) | 11.7 (9.7) | 11.8 (13.9) | 0.0071 |
| | | | | | | | |
| **Hospital market characteristics** | | | | **Unweighted** | **Weighted** | | |
| Population, mean (SD) | 2166615.7 (2090367.6) | 2329261.1 (1817851.7) | 0.083 | 2294207.1 (2084985.4) | 2270191.4 (1290210.7) | 2309094.7 (1818995.0) | 0.0247 |
| Admissions from low-income areas**, mean (SD) | 49.0 (22.2) | 46.1 (23.4) | -0.1263 | 48.9 (22.5) | 48.4 (14.3) | 46.3 (23.5) | -0.1071 |
| MA penetration, mean (SD) | 35.3 (13.3) | 33.4 (10.9) | -0.1551 | 32.9 (12.1) | 32.8 (7.7) | 33.5 (10.8) | 0.0753 |
| SNF beds per 10000 patients, mean (SD) | 10987.4 (9432.0) | 11438.9 (9635.6) | 0.0474 | 11466.1 (9265.6) | 11424.0 (5805.5) | 11368.3 (9679.7) | -0.007 |

© 2025 Crowley AP et al. *JAMA Health Forum.*

| | | | | | | | |
|---|---|---|---|---|---|---|---|
| Hospital Concentration***, mean (SD) | 1897.0 (1626.9) | 1592.7 (1205.9) | -0.2125 | 1598.6 (1308.9) | 1564.7 (800.1) | 1607.5 (1210.2) | 0.0418 |

*Medicare days as a proportion of total inpatient days
** Defined as ZIP codes in which median household income <$40,000
***HHI is a measure of market concentration that ranges from 0 to 10,000, with higher values corresponding to greater concentration

**eTable 2. Characteristics of Physicians in BPCI Advanced and Non-BPCI Advanced PGPs, Before and After Propensity Score Matching**

Physicians that had ever (BPCI-A ever) or never (BPCI-A never) practiced in a PGP that participated in BPCI Advanced were matched according to the 3:1 propensity score matching procedure detailed above in eMethods 6. Standardized mean differences (SMD) within +/- 0.05 suggest that our matching procedure performed well.

| | Before matching | | | After matching | | | |
|---|---|---|---|---|---|---|---|
| | BPCI-A never | BPCI-A ever | SMD | BPCI-A never | | BPCI-A ever | SMD |
| **Total no. of NPIs** | 11079 | 2950 | | 4671 | | 2820 | |
| | | | | | | | |
| **Physician characteristics** | | | | **Unweighted** | **Weighted** | | |
| Age, mean (SD) | 49.3 (10.8) | 48.3 (9.8) | -0.0981 | 48.8 (10.1) | 48.4 (7.7) | 48.5 (9.8) | 0.0128 |
| Male, N (%) | 10165 (91.8) | 2820 (95.6) | -0.1583 | 4501 (96.4) | 2737 (97.1) | 2737 (97.1) | 0 |
| Annual services provided, mean (SD) | 1385.1 (1322.8) | 1790.4 (1236.9) | 0.3165 | 1679.3 (1456.7) | 1821.2 (1246.9) | 1821.3 (1225.6) | 0.0001 |
| Payment for annual services, mean (SD) | 180708.0 (145797.6) | 230326.9 (152813.0) | 0.3322 | 218766.1 (162263.0) | 238746.0 (142740.1) | 235010.1 (151777.3) | -0.0254 |
| Beneficiaries cared for, mean (SD) | 380.2 (277.9) | 471.2 (285.2) | 0.3231 | 442.4 (276.7) | 478.6 (235.7) | 477.3 (277.5) | -0.005 |
| Proportion of services provided in the inpatient setting, mean (SD) | 15.5 (20.2) | 10.3 (13.0) | -0.3039 | 11.4 (14.1) | 10.2 (9.3) | 10.0 (11.9) | -0.0211 |

© 2025 Crowley AP et al. *JAMA Health Forum.*

| | | | | | | | |
|---|---|---|---|---|---|---|---|
| Relative proportion of services provided in the outpatient setting*, N (%) | 2087 (19.3) | 175 (6.0) | -0.4085 | 395 (8.5) | 150 (5.3) | 156 (5.5) | 0.0094 |
| CBSA type, N (%) | | | 0.3536 | | | | 0 |
| Metropolitan | 9544 (88.4) | 2698 (92.7) | | 4266 (91.3) | 2617 (92.8) | 2617 (92.8) | |
| Micropolitan | 980 (9.1) | 164 (5.6) | | 336 (7.2) | 158 (5.6) | 158 (5.6) | |
| Rural | 251 (2.3) | 47 (1.6) | | 69 (1.5) | 45 (1.6) | 45 (1.6) | |
| Missing CBSA | 22 (0.2) | 0 (0.0) | | | | | |
| Health system affiliated, N (%) | 9631 (86.9) | 2730 (92.5) | 0.1857 | 4393 (94.0) | 2686 (95.3) | 2679 (95.0) | -0.0121 |
| Care for patients at a teaching hospital, N (%) | 8945 (82.4) | 2483 (85.5) | 0.0843 | 3882 (83.2) | 2363 (83.9) | 2407 (85.4) | 0.0425 |
| Proportion of admissions from dual-eligible individuals, mean (SD) | 17.0 (21.4) | 12.8 (16.1) | -0.2239 | 13.9 (16.6) | 12.7 (11.7) | 12.7 (15.8) | 0.0011 |
| Proportion of admissions from Black individuals, mean (SD) | 6.3 (14.0) | 5.8 (10.7) | -0.0369 | 5.9 (12.0) | 5.6 (8.8) | 5.8 (10.4) | 0.0144 |
| Proportion of Lower extremity joint replacement admissions among BPCI-A episodes, mean (SD) | 60.5 (30.2) | 63.4 (28.8) | 0.0991 | 62.8 (28.7) | 64.1 (22.2) | 63.5 (28.7) | -0.0226 |
| Proportion of admissions accounted for by the highest volume hospital**, mean (SD) | 91.6 (15.5) | 88.5 (17.2) | -0.188 | 89.6 (17.0) | 88.5 (13.7) | 88.4 (17.3) | -0.0067 |

© 2025 Crowley AP et al. *JAMA Health Forum.*

| | | | | | | | |
|---|---|---|---|---|---|---|---|
| | | | | | | | |
| **TIN characteristics** | | | | **Unweighted** | **Weighted** | | |
| Physicians within a TIN, mean (SD) | 16.1 (22.0) | 23.8 (26.3) | 0.3174 | 19.5 (26.2) | 24.1 (24.2) | 23.8 (26.3) | -0.0115 |
| Multi-specialty TIN, N (%) | 9818 (96.9) | 2815 (99.6) | 0.211 | 4622 (99.0) | 2802 (99.4) | 2810 (99.6) | 0.0404 |
| | | | | | | | |
| **Market characteristics** | | | | **Unweighted** | **Weighted** | | |
| Population, mean (SD) | 2301891.2 (2083933.1) | 2055816.5 (1702105.4) | -0.1293 | 2079679.8 (1877905.3) | 2016644.9 (1403710.0) | 2053063.6 (1701843.7) | 0.0233 |
| Admissions from low-income areas***, mean (SD) | 45.1 (22.4) | 47.7 (22.8) | 0.1144 | 46.9 (22.8) | 47.4 (18.0) | 47.5 (22.9) | 0.0068 |
| MA penetration, mean (SD) | 35.5 (13.1) | 33.4 (11.8) | -0.1641 | 34.1 (12.6) | 33.7 (9.5) | 33.4 (11.8) | -0.0268 |
| SNF beds per 10000 patients, mean (SD) | 13765.4 (11682.6) | 12478.8 (10044.6) | -0.1181 | 12673.1 (10898.6) | 12202.8 (8189.1) | 12476.7 (10061.3) | 0.0299 |
| Hospital Concentration****, mean (SD) | 1822.4 (1612.0) | 1982.8 (1817.3) | 0.0934 | 1962.9 (1697.5) | 2019.9 (1345.2) | 1987.8 (1819.5) | -0.02 |

*Physicians with greater than the median proportion of services provided in the outpatient setting defined as higher; physicians with proportions lower than the median define as lower.
**Among all admissions by a physician to different hospitals, the proportion of admissions accounted for by the highest volume hospital.
*** Defined as ZIP codes in which median household income <$40,000
****HHI is a measure of market concentration that ranges from 0 to 10,000, with higher values corresponding to greater concentration

© 2025 Crowley AP et al. *JAMA Health Forum.*

**eTable 3. Unadjusted Changes in 90-day Outcomes, Baseline versus Intervention Period**

Unadjusted analyses of pre- and post-spending, utilization, and quality among non-participants and participating physicians and hospitals demonstrated reductions in total spending among all groups, with the greatest reduction among physicians, followed by "both," hospitals, and finally non-participants. Of note, hospital participants experienced an unadjusted increase in spending on readmissions, while physicians and non-participants experienced a decrease in spending on readmissions. Utilization of post-acute care decreased among all participant types except for an increase in home health agency use among hospitals and an increase in 7-day office visits among physicians.

| | Non BPCI-A | | | BPCI-A PGP only | | | BPCI-A HOSP only | | | BPCI-A BOTH | | |
|---|---|---|---|---|---|---|---|---|---|---|---|---|
| | **Pre** | **Post** | **Dif** | **Pre** | **Post** | **Dif** | **Pre** | **Post** | **Dif** | **Pre** | **Post** | **Dif** |
| **Spending** | | | | | | | | | | | | |
| Total episode spending, mean (SD) | 27764.3 (17210.6) | 27561.7 (17101.3) | -202.6 | 26482.7 (16247.9) | 25212.6 (15079.6) | -1270.1 | 29854.2 (18928.8) | 29234.5 (18334.3) | -619.8 | 28554.8 (17356.6) | 27671.2 (16522.5) | -883.7 |
| Index admission, Mean (SD) | 15475.2 (2063.3) | 15476.0 (2175.6) | 0.8 | 15556.2 (2518.8) | 15528.4 (2019.0) | -27.8 | 15407.0 (2170.8) | 15472.3 (2276.0) | 65.3 | 15631.6 (2113.0) | 15710.2 (2180.5) | 78.5 |
| Readmission, Mean (SD) | 1898.6 (6134.5) | 1869.2 (6145.6) | -29.4 | 1743.6 (5862.9) | 1636.9 (5759.0) | -106.6 | 2135.2 (6565.7) | 2191.7 (6791.7) | 56.5 | 1849.2 (6085.5) | 1922.9 (6501.9) | 73.8 |
| IRF, Mean (SD) | 1899.8 (8436.3) | 2071.1 (8841.4) | 171.3 | 1553.9 (7651.3) | 1225.3 (6883.8) | -328.6 | 2906.3 (10307.9) | 2534.0 (9864.4) | -372.3 | 2199.4 (9085.6) | 1892.2 (8350.4) | -307.2 |
| SNF, Mean (SD) | 3985.6 (9185.9) | 3532.7 (8477.6) | -452.9 | 3421.6 (8324.5) | 2696.9 (7276.9) | -724.7 | 4572.6 (9673.1) | 4033.6 (8931.8) | -539 | 4111.8 (8916.5) | 3364.9 (7835.6) | -746.9 |
| HHA, Mean (SD) | 2188.4 (2025.5) | 2187.2 (2058.5) | -1.2 | 1901.7 (2007.8) | 1700.8 (1965.0) | -200.9 | 2466.9 (2081.9) | 2519.6 (2076.1) | 52.7 | 2394.9 (1968.1) | 2292.2 (1920.6) | -102.7 |
| Hospital Outpatient, Mean (SD) | 799.6 (1642.3) | 829.6 (1730.5) | 30 | 713.1 (1558.1) | 726.1 (1600.6) | 13 | 833.2 (1601.9) | 875.9 (1710.5) | 42.7 | 727.2 (1577.5) | 759.3 (1704.2) | 32.1 |
| Physician, Mean (SD) | 1253.7 (1803.5) | 1337.0 (1934.1) | 83.3 | 1345.7 (1851.6) | 1452.7 (2176.1) | 107 | 1241.5 (1495.9) | 1304.5 (1398.0) | 63 | 1374.9 (1414.9) | 1477.8 (1339.7) | 102.9 |
| | | | | | | | | | | | | |
| **Utilization** | | | | | | | | | | | | |
| Any IRF use, N (%) | 18039 (5.2) | 7019 (5.7) | 0.5 | 8655 (4.3) | 2658 (3.4) | -0.9 | 4112 (8.0) | 1220 (6.9) | -1.2 | 1249 (6.0) | 399 (5.2) | -0.8 |
| Any SNF use, N (%) | 101183 (29.4) | 33382 (27.0) | -2.3 | 54567 (27.0) | 17637 (22.2) | -4.8 | 16570 (32.3) | 5340 (30.0) | -2.3 | 6719 (32.5) | 2243 (29.4) | -3.1 |

© 2025 Crowley AP et al. *JAMA Health Forum.*

| | | | | | | | | | | | | |
|---|---|---|---|---|---|---|---|---|---|---|---|---|
| Any HHA use, N (%) | 210283 (61.0) | 73947 (59.9) | -1.1 | 108560 (53.8) | 38607 (48.7) | -5.1 | 34188 (66.6) | 12046 (67.7) | 1 | 14141 (68.4) | 5124 (67.1) | -1.2 |
| Discharge to IRF, N (%) | 94881 (27.5) | 31119 (25.2) | -2.3 | 51184 (25.4) | 16439 (20.7) | -4.6 | 15434 (30.1) | 4974 (27.9) | -2.1 | 6285 (30.4) | 2090 (27.4) | -3 |
| Discharge to SNF, N (%) | 16171 (4.7) | 6222 (5.0) | 0.3 | 7686 (3.8) | 2238 (2.8) | -1 | 3708 (7.2) | 1082 (6.1) | -1.2 | 1136 (5.5) | 356 (4.7) | -0.8 |
| Discharge to HHA, N (%) | 113873 (33.1) | 39602 (32.1) | -1 | 58762 (29.1) | 21468 (27.1) | -2 | 16625 (32.4) | 6067 (34.1) | 1.7 | 7856 (38.0) | 2962 (38.8) | 0.8 |
| SNF LOS, Mean (SD) | 24.8 (24.2) | 25.3 (21.2) | 0.5 | 23.0 (20.1) | 23.2 (19.7) | 0.2 | 25.3 (20.8) | 25.4 (20.8) | 0.1 | 22.7 (19.7) | 22.0 (19.2) | -0.7 |
| HHA days, Mean (SD) | 12.0 (7.8) | 11.9 (7.8) | -0.1 | 11.5 (7.8) | 10.8 (7.7) | -0.7 | 12.8 (8.3) | 12.4 (8.1) | -0.4 | 11.7 (8.0) | 10.6 (7.6) | -1.1 |
| 7-day post-discharge physician office visit, N (%) | 39789 (11.6) | 15136 (12.3) | 0.7 | 34522 (17.1) | 16848 (21.3) | 4.2 | 4042 (7.9) | 1375 (7.7) | -0.2 | 1901 (9.2) | 697 (9.1) | -0.1 |
| | | | | | | | | | | | | |
| **Quality** | | | | | | | | | | | | |
| Readmissions, N (%) | 35447 (10.3) | 12328 (10.0) | -0.3 | 19291 (9.6) | 7070 (8.9) | -0.6 | 5799 (11.3) | 2025 (11.4) | 0.1 | 2042 (9.9) | 743 (9.8) | -0.1 |
| Mortality, N (%) | 6055 (1.8) | 2152 (1.7) | 0 | 3279 (1.6) | 1264 (1.6) | 0 | 1038 (2.0) | 380 (2.1) | 0.1 | 355 (1.7) | 133 (1.7) | 0 |
| ED visit*, N (%) | 48764 (14.2) | 17435 (14.1) | 0 | 27485 (13.6) | 10476 (13.2) | -0.4 | 7409 (14.4) | 2604 (14.6) | 0.2 | 2942 (14.2) | 1055 (13.8) | -0.4 |
| | | | | | | | | | | | | |
| Lower extremity joint replacement complications , N (%) | 13922 (4.0) | 4604 (3.7) | -0.3 | 7332 (3.6) | 2625 (3.3) | -0.3 | 2281 (4.4) | 781 (4.4) | -0.1 | 818 (4.0) | 296 (3.9) | -0.1 |
| * not leading to inpatient admission | | | | | | | | | | | | |

© 2025 Crowley AP et al. *JAMA Health Forum.*

**eTable 4. Difference-in-Differences Estimates for Changes in 90-day Outcomes Associated With Bundled Payments**
Adjusted difference-in-differences estimates suggest that participation in BPCI Advanced was associated with a significant decrease in spending on IRF, SNF, and home health for physicians but only on IRF for hospitals. Similarly, physician participation was associated with a differential decrease in SNF, IRF, and home health use, while hospital participation was only associated with a differential decrease in IRF use and an increase in home health use.

| Outcome | Effect | Estimate | Lower CI | Upper CI | P Value |
|---|---|---|---|---|---|
| **Spending** | | | | | |
| Spending, index admission | PGP-Non | -7.9 | -59.1 | 43.3 | 0.763 |
| | Hosp-Non | 39.2 | -11.9 | 90.3 | 0.133 |
| | Hosp-PGP | 47.0 | -19.6 | 113.7 | 0.166 |
| | Both-Non | 25.7 | -37.3 | 88.8 | 0.423 |
| Spending, readmissions | PGP-Non | -35.3 | -97.0 | 26.4 | 0.262 |
| | Hosp-Non | 25.3 | -104.2 | 154.9 | 0.701 |
| | Hosp-PGP | 60.6 | -78.9 | 200.1 | 0.394 |
| | Both-Non | 67.5 | -123.4 | 258.4 | 0.488 |
| Spending, IRF | PGP-Non | -473.8 | -617.2 | -330.4 | <.0001 |
| | Hosp-Non | -510.3 | -799.0 | -221.5 | 0.001 |
| | Hosp-PGP | -36.5 | -338.9 | 265.9 | 0.813 |
| | Both-Non | -553.0 | -1016.6 | -89.3 | 0.019 |
| Spending, SNF | PGP-Non | -178.1 | -285.1 | -71.0 | 0.001 |
| | Hosp-Non | -208.9 | -424.8 | 6.9 | 0.058 |
| | Hosp-PGP | -30.9 | -244.0 | 182.3 | 0.776 |
| | Both-Non | -541.1 | -899.6 | -182.5 | 0.003 |
| Spending, HHA | PGP-Non | -172.7 | -216.5 | -128.9 | <.0001 |
| | Hosp-Non | 31.3 | -26.9 | 89.4 | 0.291 |
| | Hosp-PGP | 204.0 | 138.7 | 269.2 | <.0001 |
| | Both-Non | -141.3 | -267.9 | -14.6 | 0.029 |
| | PGP-Non | -9.1 | -27.8 | 9.6 | 0.339 |
| | Hosp-Non | 7.9 | -26.0 | 41.9 | 0.646 |

© 2025 Crowley AP et al. *JAMA Health Forum.*

| | | | | | |
|---|---|---|---|---|---|
| Spending, Hospital Outpatient | Hosp-PGP | 17.0 | 18.2 | 52.3 | 0.343 |
| | Both-Non | 14.8 | -36.1 | 65.7 | 0.569 |
| Spending, Physician (Part B Carrier File) | PGP-Non | 10.9 | -14.2 | 36.0 | 0.395 |
| | Hosp-Non | -10.1 | -51.7 | 31.5 | 0.634 |
| | Hosp-PGP | -21.0 | -65.3 | 23.3 | 0.353 |
| | Both-Non | -13.1 | -65.8 | 39.6 | 0.626 |
| **Utilization** | | | | | |
| Any IRF use | PGP-Non | -1.33% | -1.73% | -0.92% | <.0001 |
| | Hosp-Non | -1.49% | -2.31% | -0.68% | <.0001 |
| | Hosp-PGP | -0.17% | -1.03% | 0.70% | 0.707 |
| | Both-Non | -1.47% | -2.77% | -0.18% | 0.026 |
| Any SNF use | PGP-Non | -1.92% | -2.71% | -1.13% | <.0001 |
| | Hosp-Non | -0.96% | -2.68% | 0.75% | 0.270 |
| | Hosp-PGP | 0.95% | -0.83% | 2.73% | 0.293 |
| | Both-Non | -2.95% | -5.58% | -0.32% | 0.028 |
| Any HHA use | PGP-Non | -3.39% | -4.65% | -2.14% | <.0001 |
| | Hosp-Non | 1.49% | 0.00% | 2.97% | 0.050 |
| | Hosp-PGP | 4.88% | 3.17% | 6.59% | <.0001 |
| | Both-Non | -1.30% | -4.94% | 2.34% | 0.484 |
| **Quality** | | | | | |
| Mortality | PGP-Non | 0.06% | -0.06% | 0.18% | 0.312 |
| | Hosp-Non | 0.06% | -0.21% | 0.32% | 0.678 |
| | Hosp-PGP | -0.01% | -0.27% | 0.26% | 0.969 |
| | Both-Non | 0.01% | -0.33% | 0.34% | 0.975 |
| Readmissions | PGP-Non | -0.15% | -0.47% | 0.17% | 0.364 |
| | Hosp-Non | 0.05% | -0.55% | 0.65% | 0.872 |
| | Hosp-PGP | 0.20% | -0.45% | 0.85% | 0.552 |
| | Both-Non | 0.10% | -0.85% | 1.04% | 0.840 |
| | PGP-Non | -0.27% | -0.64% | 0.10% | 0.149 |

© 2025 Crowley AP et al. *JAMA Health Forum.*

| | | | | | |
|---|---|---|---|---|---|
| ED visits not leading to an inpatient admission | Hosp-Non | 0.16% | -0.48% | 0.80% | 0.632 |
| | Hosp-PGP | 0.43% | -0.23% | 1.09% | 0.203 |
| | Both-Non | -0.29% | -1.17% | 0.60% | 0.525 |
| Lower extremity joint replacement complications | PGP-Non | 0.09% | -0.10% | 0.27% | 0.369 |
| | Hosp-Non | 0.05% | -0.30% | 0.41% | 0.764 |
| | Hosp-PGP | -0.03% | -0.40% | 0.33% | 0.862 |
| | Both-Non | 0.06% | -0.57% | 0.69% | 0.858 |

© 2025 Crowley AP et al. *JAMA Health Forum.*

**eTable 5. Unadjusted Changes in 30-day Outcomes, Baseline versus Intervention Period**

Unadjusted analyses of pre- and post-spending, utilization, and quality among non-participants and participating physicians and hospitals demonstrated reductions in total spending among all groups, with the greatest reduction among physicians, followed by "both," hospitals, and finally non-participants. Utilization and spending on of post-acute care decreased among all participant types except for an increase in home health agency use and spending among hospitals and an increase in 7-day office visits among physicians.

| | Non-BPCI-A | | | BPCI-A PGP | | | BPCI-A HOSP | | | BPCI-A BOTH | | |
|---|---|---|---|---|---|---|---|---|---|---|---|---|
| | **Pre** | **Post** | **Dif** | **Pre** | **Post** | **Dif** | **Pre** | **Post** | **Dif** | **Pre** | **Post** | **Dif** |
| **Spending** | | | | | | | | | | | | |
| Total spending, mean (SD) | 24371.0 (13248.7) | 24197.3 (13290.0) | -173.7 | 23360.9 (12534.9) | 22239.6 (11544.3) | -1121.3 | 26153.3 (14873.1) | 25481.1 (14155.0) | -672.3 | 25082.4 (13379.8) | 24311.7 (12754.1) | -770.7 |
| Index admission, Mean (SD) | 15475.2 (2063.3) | 15476.0 (2175.6) | 0.8 | 15556.2 (2518.8) | 15528.4 (2019.0) | -27.8 | 15407.0 (2170.8) | 15472.3 (2276.0) | 65.3 | 15631.6 (2113.0) | 15710.2 (2180.5) | 78.5 |
| Readmission, Mean (SD) | 876.1 (3732.5) | 866.8 (3759.2) | -9.2 | 820.6 (3626.7) | 783.0 (3581.0) | -37.5 | 1027.6 (4080.9) | 1018.5 (4101.1) | -9.2 | 830.8 (3652.5) | 916.3 (3904.0) | 85.4 |
| IRF, Mean (SD) | 1772.5 (7947.4) | 1925.3 (8265.6) | 152.8 | 1456.9 (7251.2) | 1131.9 (6466.0) | -324.9 | 2743.8 (9788.1) | 2368.1 (9272.3) | -375.7 | 2070.1 (8528.8) | 1779.6 (7898.2) | -290.5 |
| SNF, Mean (SD) | 3570.1 (8052.4) | 3205.1 (7536.2) | -365 | 3067.6 (7266.3) | 2439.8 (6439.6) | -627.8 | 4092.9 (8429.8) | 3636.6 (7872.6) | -456.3 | 3714.7 (7822.0) | 3043.3 (6921.9) | -671.4 |
| HHA, Mean (SD) | 1885.7 (1779.5) | 1880.1 (1804.3) | -5.5 | 1640.6 (1754.8) | 1460.8 (1702.5) | -179.8 | 2097.7 (1817.8) | 2159.7 (1810.2) | 62 | 2062.0 (1704.6) | 2007.0 (1679.1) | -55 |
| Hospital Outpatient, Mean (SD) | 257.4 (649.2) | 260.4 (665.5) | 3.1 | 239.0 (668.3) | 239.8 (626.6) | 0.9 | 260.9 (644.8) | 258.7 (661.0) | -2.2 | 214.0 (580.3) | 236.8 (661.1) | 22.8 |
| Physician, Mean (SD) | 400.9 (649.3) | 457.0 (836.1) | 56.2 | 458.7 (695.4) | 533.4 (991.2) | 74.8 | 375.7 (532.2) | 418.4 (590.9) | 42.7 | 431.0 (521.7) | 498.9 (589.1) | 67.9 |
| | | | | | | | | | | | | |
| **Utilization** | | | | | | | | | | | | |
| Any IRF use, N (%) | 17269 (5.0) | 6717 (5.4) | 0.4 | 8286 (4.1) | 2510 (3.2) | -0.9 | 3970 (7.7) | 1171 (6.6) | -1.2 | 1215 (5.9) | 385 (5.0) | -0.8 |
| Any SNF use, N (%) | 99617 (28.9) | 32805 (26.6) | -2.3 | 53739 (26.6) | 17330 (21.9) | -4.8 | 16334 (31.8) | 5235 (29.4) | -2.4 | 6618 (32.0) | 2195 (28.8) | -3.2 |
| Any HHA use, N (%) | 194132 (56.4) | 68203 (55.3) | -1.1 | 100397 (49.7) | 35713 (45.0) | -4.7 | 31295 (61.0) | 11122 (62.5) | 1.5 | 13125 (63.5) | 4801 (62.9) | -0.6 |

© 2025 Crowley AP et al. *JAMA Health Forum.*

| | | | | | | | | | | | | |
|---|---|---|---|---|---|---|---|---|---|---|---|---|
| Discharge to IRF, N (%) | 94881 (27.5) | 31119 (25.2) | -2.3 | 51184 (25.4) | 16439 (20.7) | -4.6 | 15434 (30.1) | 4974 (27.9) | -2.1 | 6285 (30.4) | 2090 (27.4) | -3 |
| Discharge to SNF, N (%) | 16171 (4.7) | 6222 (5.0) | 0.3 | 7686 (3.8) | 2238 (2.8) | -1 | 3708 (7.2) | 1082 (6.1) | -1.2 | 1136 (5.5) | 356 (4.7) | -0.8 |
| Discharge to HHA, N (%) | 113873 (33.1) | 39602 (32.1) | -1 | 58762 (29.1) | 21468 (27.1) | -2 | 16625 (32.4) | 6067 (34.1) | 1.7 | 7856 (38.0) | 2962 (38.8) | 0.8 |
| SNF LOS, Mean (SD) | 22.7 (21.6) | 23.2 (18.2) | 0.5 | 21.0 (17.1) | 21.2 (16.8) | 0.2 | 23.1 (17.7) | 23.2 (17.9) | 0 | 20.9 (16.9) | 20.2 (16.5) | -0.7 |
| HHA days, Mean (SD) | 10.9 (5.7) | 10.8 (5.7) | -0.1 | 10.4 (5.8) | 9.6 (5.7) | -0.8 | 11.5 (6.0) | 11.2 (5.9) | -0.3 | 10.5 (5.7) | 9.6 (5.6) | -1 |
| 7-day post-discharge physician office visit, N (%) | 39789 (11.6) | 15136 (12.3) | 0.7 | 34522 (17.1) | 16848 (21.3) | 4.2 | 4042 (7.9) | 1375 (7.7) | -0.2 | 1901 (9.2) | 697 (9.1) | -0.1 |
| | | | | | | | | | | | | |
| **<u>Quality</u>** | | | | | | | | | | | | |
| Mortality, N (%) | 2736 (0.8) | 955 (0.8) | 0 | 1552 (0.8) | 567 (0.7) | -0.1 | 469 (0.9) | 168 (0.9) | 0 | 160 (0.8) | 69 (0.9) | 0.1 |
| Readmissions, N (%) | 17958 (5.2) | 6246 (5.1) | -0.2 | 9957 (4.9) | 3741 (4.7) | -0.2 | 3050 (6.0) | 1038 (5.8) | -0.1 | 999 (4.8) | 402 (5.3) | 0.4 |
| ED visit*, N (%) | 28107 (8.2) | 9966 (8.1) | -0.1 | 15879 (7.9) | 6029 (7.6) | -0.3 | 4226 (8.2) | 1465 (8.2) | 0 | 1647 (8.0) | 657 (8.6) | 0.6 |
| | | | | | | | | | | | | |
| Lower extremity joint replacement complications, N (%) | 11898 (3.5) | 3884 (3.1) | -0.3 | 6295 (3.1) | 2236 (2.8) | -0.3 | 1941 (3.8) | 666 (3.7) | 0 | 690 (3.3) | 256 (3.4) | 0 |

© 2025 Crowley AP et al. *JAMA Health Forum.*

**eTable 6. Difference-in-Differences Estimates for Changes in 30-day Outcomes Associated With Bundled Payments**
Adjusted difference-in-differences estimates suggest that participation in BPCI Advanced was associated with a significant decrease in 30-day spending on IRF, SNF, and home health for physicians but only on IRF for hospitals. Similarly, physician participation was associated with a differential decrease in SNF, IRF, and home health use, while hospital participation was only associated with a differential decrease in IRF use and an increase in home health use.

| Outcome | Effect | Estimate | Lower CI | Upper CI | P Value |
|---|---|---|---|---|---|
| **Spending** | | | | | |
| Total spending, mean (SD) | PGP-Non | -785.3 | -974.5 | -596.1 | <.0001 |
| | Hosp-Non | -647.9 | -1022.8 | -273.1 | 0.001 |
| | Hosp-PGP | 137.3 | -276.5 | 551.2 | 0.515 |
| | Both-Non | -1006.9 | -1607.2 | -406.6 | 0.001 |
| Spending, index admission | PGP-Non | -7.9 | -59.1 | 43.3 | 0.763 |
| | Hosp-Non | 39.2 | -11.9 | 90.3 | 0.133 |
| | Hosp-PGP | 47.0 | -19.6 | 113.7 | 0.166 |
| | Both-Non | 25.7 | -37.3 | 88.8 | 0.423 |
| Spending, readmissions | PGP-Non | -10.8 | -51.5 | 29.8 | 0.601 |
| | Hosp-Non | -32.3 | -108.8 | 44.2 | 0.407 |
| | Hosp-PGP | -21.5 | -108.9 | 65.9 | 0.630 |
| | Both-Non | 70.7 | -35.7 | 177.0 | 0.193 |
| Spending, IRF | PGP-Non | -452.3 | -590.9 | -313.6 | <.0001 |
| | Hosp-Non | -496.0 | -773.5 | -218.4 | <.0001 |
| | Hosp-PGP | -43.7 | -340.2 | 252.9 | 0.773 |
| | Both-Non | -516.2 | -964.5 | -67.8 | 0.024 |
| Spending, SNF | PGP-Non | -182.3 | -282.3 | -82.3 | <.0001 |
| | Hosp-Non | -201.3 | -405.6 | 2.9 | 0.053 |
| | Hosp-PGP | -19.0 | -217.9 | 179.8 | 0.851 |
| | Both-Non | -535.3 | -867.9 | -202.7 | 0.002 |
| Spending, HHA | PGP-Non | -155.6 | -196.6 | -114.6 | <.0001 |
| | Hosp-Non | 48.6 | -4.4 | 101.5 | 0.072 |

© 2025 Crowley AP et al. *JAMA Health Forum.*

| | | | | | |
|---|---|---|---|---|---|
| | Hosp-PGP | 204.2 | 145.4 | 263.0 | <.0001 |
| | Both-Non | -83.7 | -203.8 | 36.4 | 0.172 |
| Spending, Hospital outpatient | PGP-Non | 1.1 | -7.2 | 9.3 | 0.802 |
| | Hosp-Non | -4.3 | -18.1 | 9.5 | 0.545 |
| | Hosp-PGP | -5.3 | -20.1 | 9.4 | 0.479 |
| | Both-Non | 25.7 | 3.7 | 47.7 | 0.022 |
| Spending, Physician (Part B Carrier File) | PGP-Non | 10.8 | -4.7 | 26.3 | 0.172 |
| | Hosp-Non | -9.4 | -32.3 | 13.4 | 0.418 |
| | Hosp-PGP | -20.2 | -44.3 | 3.8 | 0.100 |
| | Both-Non | 6.4 | -22.5 | 35.3 | 0.665 |
| **Utilization** | | | | | |
| Any IRF use | PGP-Non | -1.31% | -1.70% | -0.91% | <.0001 |
| | Hosp-Non | -1.47% | -2.29% | -0.66% | <.0001 |
| | Hosp-PGP | -0.16% | -1.03% | 0.70% | 0.710 |
| | Both-Non | -1.47% | -2.77% | -0.16% | 0.028 |
| Any SNF use | PGP-Non | -1.89% | -2.68% | -1.10% | <.0001 |
| | Hosp-Non | -1.08% | -2.81% | 0.65% | 0.221 |
| | Hosp-PGP | 0.81% | -0.98% | 2.60% | 0.376 |
| | Both-Non | -3.06% | -5.67% | -0.45% | 0.022 |
| Any HHA use | PGP-Non | -3.17% | -4.42% | -1.93% | <.0001 |
| | Hosp-Non | 1.98% | 0.45% | 3.51% | 0.011 |
| | Hosp-PGP | 5.16% | 3.42% | 6.89% | <.0001 |
| | Both-Non | -0.45% | -4.09% | 3.20% | 0.810 |
| Discharge to IRF | PGP-Non | -1.27% | -1.65% | -0.88% | <.0001 |
| | Hosp-Non | -1.37% | -2.16% | -0.58% | 0.001 |
| | Hosp-PGP | -0.11% | -0.95% | 0.74% | 0.804 |
| | Both-Non | -1.39% | -2.66% | -0.12% | 0.031 |
| Discharge to SNF | PGP-Non | -1.77% | -2.57% | -0.96% | <.0001 |
| | Hosp-Non | -0.77% | -2.52% | 0.98% | 0.391 |

© 2025 Crowley AP et al. *JAMA Health Forum.*

| | | | | | |
|---|---|---|---|---|---|
| | Hosp-PGP | 1.00% | -0.80% | 2.81% | 0.277 |
| | Both-Non | -2.80% | -5.48% | -0.13% | 0.040 |
| Discharge to HHA | PGP-Non | -0.95% | -2.20% | 0.31% | 0.139 |
| | Hosp-Non | 2.84% | 0.70% | 4.98% | 0.009 |
| | Hosp-PGP | 3.79% | 1.54% | 6.03% | 0.001 |
| | Both-Non | 1.47% | -2.80% | 5.73% | 0.500 |
| SNF Length of Stay | PGP-Non | -0.8 | -1.2 | -0.4 | <.0001 |
| | Hosp-Non | -0.7 | -1.2 | -0.1 | 0.017 |
| | Hosp-PGP | 0.1 | -0.5 | 0.7 | 0.781 |
| | Both-Non | -1.4 | -2.3 | -0.5 | 0.003 |
| HHA days | PGP-Non | -0.8 | -1.0 | -0.6 | <.0001 |
| | Hosp-Non | -0.2 | -0.5 | 0.1 | 0.154 |
| | Hosp-PGP | 0.6 | 0.3 | 0.9 | <.0001 |
| | Both-Non | -0.9 | -1.4 | -0.4 | <.0001 |
| 7-day physician office visits | PGP-Non | 2.88% | 1.98% | 3.79% | <.0001 |
| | Hosp-Non | -0.48% | -1.44% | 0.48% | 0.324 |
| | Hosp-PGP | -3.37% | -4.51% | -2.22% | <.0001 |
| | Both-Non | 0.58% | -1.40% | 2.56% | 0.566 |
| **Quality** | | | | | |
| Mortality, N (%) | PGP-Non | 0.00% | -0.08% | 0.09% | 0.950 |
| | Hosp-Non | 0.01% | -0.18% | 0.19% | 0.931 |
| | Hosp-PGP | 0.01% | -0.19% | 0.20% | 0.956 |
| | Both-Non | 0.13% | -0.12% | 0.37% | 0.318 |
| Readmissions, N (%) | PGP-Non | 0.02% | -0.22% | 0.27% | 0.847 |
| | Hosp-Non | -0.17% | -0.58% | 0.25% | 0.424 |
| | Hosp-PGP | -0.19% | -0.65% | 0.27% | 0.411 |
| | Both-Non | 0.49% | -0.12% | 1.11% | 0.117 |
| ED visits not leading to an inpatient admission | PGP-Non | -0.12% | -0.41% | 0.18% | 0.439 |
| | Hosp-Non | 0.06% | -0.43% | 0.56% | 0.798 |
| | Hosp-PGP | 0.18% | -0.34% | 0.71% | 0.497 |

© 2025 Crowley AP et al. *JAMA Health Forum.*

| | | | | | |
|---|---|---|---|---|---|
| | Both-Non | 0.82% | 0.11% | 1.52% | 0.023 |
| Lower extremity joint replacement complications | PGP-Non | 0.09% | -0.07% | 0.26% | 0.277 |
| | Hosp-Non | 0.08% | -0.26% | 0.43% | 0.633 |
| | Hosp-PGP | -0.01% | -0.36% | 0.34% | 0.967 |
| | Both-Non | 0.13% | -0.40% | 0.66% | 0.625 |

© 2025 Crowley AP et al. *JAMA Health Forum.*

**eTables 7-11. Sensitivity Analyses.**
All five sensitivity analyses shown below demonstrate findings that are roughly equivalent in magnitude to those with our main model specification. This suggests that our results are robust to changes in matching procedure, program specifics, and anticipation effects.

**eTable 7. Sensitivity Analysis, 1:1 Matching**

| Outcome | Effect | Estimate | Lower CI | Upper CI | P Value |
|---|---|---|---|---|---|
| Total episode spending, 90 days | PGP-Non | -842.5 | -1014.9 | -670.1 | <.0001 |
| | Hosp-Non | -499.1 | -774.3 | -223.9 | 0.0004 |
| | Hosp-PGP | 343.4 | 74.9 | 611.9 | 0.0122 |
| | Both-Non | -1068.5 | -1454.5 | -682.5 | <.0001 |

**eTable 8. Sensitivity Analysis, Treating "Both" group as PGP**

| Outcome | Effect | Estimate | Lower CI | Upper CI | P Value |
|---|---|---|---|---|---|
| Total episode spending, 90 days | PGP-Non | -880.6 | -1025.1 | -736.2 | <.0001 |
| | Hosp-Non | -588.6 | -847.4 | -329.8 | <.0001 |
| | Hosp-PGP | 292.0 | 25.8 | 558.2 | 0.0316 |

**eTable 9. Sensitivity Analysis, Overall Program Effect**

| Outcome | Effect | Estimate | Lower CI | Upper CI | P Value |
|---|---|---|---|---|---|
| Total episode spending, 90 days | BPCI-A vs Non | -839.0 | -975.7 | -702.2 | <.0001 |

**eTable 10. Sensitivity Analysis, Washout Period**
Removed quarters 2 and 3 of 2018 (April – September)

| Outcome | Effect | Estimate | Lower CI | Upper CI | P Value |
|---|---|---|---|---|---|
| Total episode spending, 90 days | PGP-Non | -894.7 | -1047.9 | -741.6 | <.0001 |
| | Hosp-Non | -610.1 | -876.1 | -344.1 | <.0001 |
| | Hosp-PGP | 284.6 | 8.4 | 560.7 | 0.0434 |
| | Both-Non | -1277.6 | -1674.1 | -881.1 | <.0001 |

© 2025 Crowley AP et al. *JAMA Health Forum.*

| | | | | | |
|---|---|---|---|---|---|
| Total episode spending, 30 days | PGP-Non | -835.9 | -951.1 | -720.6 | <.0001 |
| | Hosp-Non | -662.5 | -862.7 | -462.4 | <.0001 |
| | Hosp-PGP | 173.3 | -34.4 | 381.1 | 0.102 |
| | Both-Non | -1110.6 | -1408.9 | -812.2 | <.0001 |

**eTable 11. Sensitivity Analysis, Separate Participant Effects.**
PGP episodes sample: removing episodes under BPCI-A hospitals.
Hospital episodes sample: remove episodes under BPCI-A physicians.

| Outcome | Effect | Estimate | Lower CI | Upper CI | P Value |
|---|---|---|---|---|---|
| Total episode spending, 90 days | PGP-Non | -857.5 | -1104.5 | -710.5 | <.0001 |
| | Hosp-Non | -650.0 | -916.6 | -383.5 | <.0001 |

**eTable 12. Sensitivity Analysis, No Prior BPCI Advanced Participation**
We retained hospitals in the control group that participated in BPCI Advanced for other conditions besides LEJR to mitigate any effect due to selection on general value-based payment ability. However, to ensure this decision did not meaningfully change our results, we re-ran our primary analysis restricting our control group to hospitals that did not participate in BPCI Advanced at all (i.e., dropping those that participated in other conditions besides LEJR from the control group). This removed 166 hospitals (38%) and 5 physicians (0.1%) (Tables 12.1). This resulted in a 5% reduction in episodes initiated by non-participants and a 2% reduction in episodes initiated by hospital participants (Table 12.2). Our results remained robust to this change (Table 12.3).

**Table 12.1. Number of providers removed**
**12.1.1 Hospitals**

| BPCI_A_LEJR_EVER | | BPCI_A_OVERALL_EVER | | |
|---|---|---|---|---|
| | | No | Yes | Total |
| No | | | | |
| | n | 266 | 166 | 432 |
| | row % | 61.57 | 38.43 | |
| Yes | | | | |
| | n | 0 | 174 | 174 |
| | row % | 0 | 100 | |

© 2025 Crowley AP et al. *JAMA Health Forum.*

* 166 control hospitals were removed due to participating in BPCI-A for other service line groups.
* 7 treated hospitals were also removed since all their controls were removed.

**12.1.2 Physicians**

| BPCI_A_LEJR_EVER | BPCI_A_OVERALL_EVER | | Total |
|---|---|---|---|
| | No | Yes | |
| No | | | |
| n | 4666 | 5 | 4671 |
| row % | 99.89 | 0.11 | |
| Yes | | | |
| n | 0 | 2820 | 2820 |
| row % | 0 | 100 | |

* 5 control physicians were removed due to participating in BPCI-A for other service line groups.
* NO treated physicians were removed.

**Table 12.2. Number of episodes before and after the removal**

| BPCI-A LEJR GROUP | Before | After | reduction |
|---|---|---|---|
| Never | 467,924 | 445,360 | 0.05 |
| PGP only | 281,189 | 281,013 | |
| Hosp only | 69,107 | 67,653 | |
| Both | 28,309 | 28,307 | |
| Total | 846,529 | 822,333 | 0.03 |

**Table 12.3. Main model results after removal**

**Outcome: 90-d total episode spending**

| Effect | Estimate | LowerCI | UpperCI | P value |
|---|---|---|---|---|
| PGP-Non at T1-T0 | -845.87 | -994.93 | -696.80 | <.0001 |
| Hosp-Non at T1-T0 | -613.69 | -874.02 | -353.35 | <.0001 |
| Hosp-PGP at T1-T0 | 232.18 | -36.91 | 501.26 | 0.0908 |
| Both-Non at T1-T0 | -1139.68 | -1522.62 | -756.73 | <.0001 |

© 2025 Crowley AP et al. *JAMA Health Forum.*

**eTable 13. Exploratory Analysis, Differential Changes in Patient Characteristics**

Changes in patient characteristics were measured with each of Elixhauser score, dual-eligibility, disability, prior SNF use, and prior hospital use as the dependent variable and all remaining variables from the main model as covariates. Adjusted estimates suggest a non-significant trend toward lower Elixhauser score in the post-period among physicians in participating PGPs and significant differences in prior SNF and hospital use among patients treated by participating physicians.

| | Parameter | Estimate | 95% Confidence Limits | | P Value |
|---|---|---|---|---|---|
| Elixhauser | PGP Only | -0.0973 | -0.195 | 0.0003 | 0.0507 |
| | Hospital Only | -0.0234 | -0.1935 | 0.1468 | 0.7876 |
| Dual-Eligible | PGP Only | -0.0017 | -0.0044 | 0.001 | 0.2222 |
| | Hospital Only | -0.0002 | -0.0049 | 0.0046 | 0.9414 |
| Disabled | PGP Only | 0.0003 | -0.0017 | 0.0024 | 0.7428 |
| | Hospital Only | -0.0018 | -0.0054 | 0.0018 | 0.3227 |
| Prior SNF | PGP Only | 0.0021 | 0 | 0.0041 | 0.0469 |
| | Hospital Only | 0.0004 | -0.0032 | 0.004 | 0.8301 |
| Prior Hosp | PGP Only | -0.0149 | -0.019 | -0.0108 | <.0001 |
| | Hospital Only | -0.0015 | -0.0086 | 0.0056 | 0.6835 |

**eTable 14. Exploratory Analyses, Unadjusted Changes in 31-90 Day Outcomes.**

Examining unadjusted changes in outcomes during the 31–90-day post-discharge window allows us to separate the first 30 days of the episode from the rest of the 90 days. Of note, participating hospitals demonstrate an unadjusted increase in spending on readmissions in the post-discharge period, while other participant types do not.

| | Non-BPCI-A | | | BPCI-A PGP | | | BPCI-A ACH | | | BPCI-A BOTH | | |
|---|---|---|---|---|---|---|---|---|---|---|---|---|
| | Pre | Post | Dif | Pre | Post | Dif | Pre | Post | Dif | Pre | Post | Dif |
| **Spending** | | | | | | | | | | | | |
| Total 31-90-day spending, mean (SD) | 3393.3 (8005.9) | 3364.4 (7733.5) | -28.9 | 3121.8 (7475.9) | 2973.0 (6966.2) | -148.8 | 3700.9 (8390.1) | 3753.4 (8342.6) | 52.5 | 3472.4 (7939.1) | 3359.5 (7599.5) | -113 |
| Readmission, Mean (SD) | 1022.5 (4343.5) | 1002.3 (4295.0) | -20.2 | 923.0 (4107.1) | 853.9 (3992.2) | -69.1 | 1107.6 (4538.6) | 1173.3 (4797.6) | 65.7 | 1018.3 (4368.6) | 1006.7 (4423.7) | -11.7 |

© 2025 Crowley AP et al. *JAMA Health Forum.*

| | | | | | | | | | | | | |
|---|---|---|---|---|---|---|---|---|---|---|---|---|
| IRF, Mean (SD) | 127.3 (2213.3) | 145.7 (2406.7) | 18.4 | 97.1 (1916.1) | 93.4 (1911.2) | -3.6 | 162.6 (2500.8) | 165.9 (2520.1) | 3.3 | 129.3 (2271.5) | 112.6 (2247.0) | -16.7 |
| SNF, Mean (SD) | 415.5 (3157.0) | 327.6 (2643.9) | -87.9 | 354.0 (2880.9) | 257.1 (2328.3) | -96.9 | 479.7 (3346.2) | 397.0 (2902.0) | -82.7 | 397.1 (3008.3) | 321.6 (2638.6) | -75.5 |
| HHA, Mean (SD) | 302.8 (1068.1) | 307.1 (1086.5) | 4.3 | 261.1 (1000.3) | 240.0 (972.7) | -21.1 | 369.2 (1173.5) | 359.9 (1161.9) | -9.3 | 333.0 (1122.5) | 285.2 (1037.3) | -47.7 |
| Hospital Outpatient, Mean (SD) | 542.2 (1406.4) | 569.1 (1481.4) | 26.9 | 474.1 (1313.5) | 486.2 (1363.8) | 12.1 | 572.4 (1365.0) | 617.2 (1480.8) | 44.8 | 513.2 (1371.6) | 522.5 (1492.0) | 9.3 |
| Physician, Mean (SD) | 852.8 (1548.9) | 880.0 (1600.9) | 27.2 | 887.0 (1579.3) | 919.3 (1715.4) | 32.2 | 865.8 (1221.3) | 886.1 (1125.3) | 20.3 | 943.9 (1179.9) | 978.9 (1033.7) | 35 |
| | | | | | | | | | | | | |
| **Utilization** | | | | | | | | | | | | |
| SNF LOS, Mean (SD) | 31.3 (20.9) | 30.4 (19.6) | -0.9 | 30.4 (20.3) | 29.4 (19.4) | -1 | 31.2 (20.0) | 29.4 (19.7) | -1.8 | 30.0 (19.5) | 28.1 (19.7) | -1.8 |
| HHA days, Mean (SD) | 14.7 (8.6) | 14.9 (8.5) | 0.2 | 14.7 (8.6) | 14.9 (8.6) | 0.2 | 15.3 (8.9) | 14.9 (8.4) | -0.4 | 15.0 (9.0) | 14.3 (8.6) | -0.8 |
| Any IRF use, N (%) | 1210 (0.4) | 489 (0.4) | 0 | 548 (0.3) | 205 (0.3) | 0 | 229 (0.4) | 82 (0.5) | 0 | 71 (0.3) | 22 (0.3) | -0.1 |
| Any SNF use, N (%) | 7977 (2.3) | 2744 (2.2) | -0.1 | 4072 (2.0) | 1407 (1.8) | -0.2 | 1340 (2.6) | 487 (2.7) | 0.1 | 463 (2.2) | 176 (2.3) | 0.1 |
| Any HHA use, N (%) | 27904 (8.1) | 9812 (7.9) | -0.2 | 14075 (7.0) | 4902 (6.2) | -0.8 | 5052 (9.8) | 1670 (9.4) | -0.5 | 1842 (8.9) | 590 (7.7) | -1.2 |
| | | | | | | | | | | | | |
| **Quality** | | | | | | | | | | | | |
| Mortality, N (%) | 3319 (1.0) | 1197 (1.0) | 0 | 1727 (0.9) | 697 (0.9) | 0 | 569 (1.1) | 212 (1.2) | 0.1 | 195 (0.9) | 64 (0.8) | -0.1 |
| Readmissions, N (%) | 20404 (5.9) | 7162 (5.8) | -0.1 | 10906 (5.4) | 3933 (5.0) | -0.4 | 3256 (6.4) | 1170 (6.6) | 0.2 | 1215 (5.9) | 427 (5.6) | -0.3 |
| ED visit*, N (%) | 24720 (7.2) | 8938 (7.2) | 0.1 | 13762 (6.8) | 5295 (6.7) | -0.1 | 3820 (7.4) | 1364 (7.7) | 0.2 | 1543 (7.5) | 483 (6.3) | -1.1 |

© 2025 Crowley AP et al. *JAMA Health Forum.*
