# Supplementary material for: Physician and Hospital Performance in Medicare’s Updated Bundled-Payment Model for Joint Replacement
Source: JAMA Health Forum. 2025 Jul 25;6(7):e251930. doi: 10.1001/jamahealthforum.2025.1930 (PMC12767874; doi:10.1001/jamahealthforum.2025.1930)
Supplement: Supplement 2. — Data Sharing Statement [file jamahealthforum-e251930-s002.pdf]

Data Sharing Statement

Crowley. Physician and Hospital Performance in Medicare's Updated Bundled-Payment Model for Joint Replacement. *JAMA Health Forum*. Published July 25, 2025. doi:10.1001/jamahealthforum.2025.1930

Data
Data available: No

Additional Information
Explanation for why data not available: Medicare claims data cannot be made publicly available.
